# Built-environment stocks in the context of a master-planned city: A case study of Chandigarh, India

**DOI:** 10.1111/jiec.13466

**Published:** 2024-04-05

**Authors:** William Mihkelson, Hadi Arbabi, Stephen Hincks, Danielle Densley Tingley

**Affiliations:** 1 Department of Civil and Structural Engineering, University of Sheffield, 412, Scalby Road, Scarborough, North Yorkshire, Sheffield, YO12 6ED England UK; 2 Department of Urban Studies and Planning, University of Sheffield, Sheffield, UK

**Keywords:** built environment, industrial ecology, material flow analysis, resource efficiency, urban planning, urbanization

## Abstract

Construction materials accumulate in the built environment forming material stocks (MS) of buildings and infrastructure, providing various services to society that result in a nexus of human development and environmental impact. Meanwhile, unprecedented urbanization in the Global South is set to put significant demand on the resources required to ensure adequate standards of living in new and existing urban areas. This is particularly important within India; however, no study has yet explored MS within cities in India or within master-planned urban areas designed to accommodate urbanization and a high standard of living. The present study begins to fill these gaps and aims to investigate patterns of built-environment MS accumulation in Chandigarh, an exceptionally quickly developed city master planned to ensure universally high standards of living through a unique urban form. We adopt a bottom-up approach to quantify the residential building and road MS at the city and sub-city scale. The results reveal that the master plan, while enabling high standards of living, has resulted in a relative accumulation of road-to-building stock that is significantly larger than in other cities. This is shown to be environmentally detrimental as future urban development is limited and promotes the demolition of existing stocks, whose composition severely limits their potential as secondary resources. The study therefore provides empirical evidence to support the integration of material stock assessments into urban planning and development to ensure resource-efficient provisioning of key services.

**Supplementary Information:**

The online version of this article (doi:10.1111/jiec.13466) contains supplementary material, which is available to authorized users.

## INTRODUCTION

Construction materials are among the most widely used materials globally, the manufacture of which accounts for 11% of process-related carbon dioxide emissions (IEA, 2019). These materials accumulate within cities in the form of built-environment material stocks (MS) which shape future material and energy use (Krausmann et al., 2017) and play important roles in society through the provision of residence, transportation, and various other services (Tanikawa et al., 2015). Built-environment MS therefore result in the nexus of anthropogenic carbon emissions and human development (Haberl et al., 2017; Müller, 2006; Müller et al., 2013; Tanikawa et al., 2015; Wiedenhofer et al., 2015), the decoupling of which is seen as critical for achieving levels of development that are considered “sustainable.” However, unprecedented urbanization in the Global South (UN, 2018) is putting significant strain on the primary resources required for built-environment MS and may threaten the achievement of sustainable development globally. More than two-thirds of the world’s population will live in cities by 2050 (UN, 2018) with around 60% of the cities required to accommodate this urbanization yet to be built (UNEP, 2013). Further, deficits in living standards (UNDP, 2019; UN-Habitat, 2020) mean nations in the Global South must increase net resource consumption to build, maintain, and upgrade the built environment to improve living standards (Krausmann et al., 2017) while simultaneously reducing environmental impact (UNEP, 2015). Built-environment MS have therefore become of increasing focus in assessments of sustainable development and resource management strategies.

### Built-environment material stocks

Built-environment MS have long lifespans, which is important to ensure continued service provision; however, this presents numerous challenges to resource efficiency and urban development. First, the legacies of such MS present lock-ins to future resource use and settlement patterns (Gutiérrez & Kahhat, 2022; Haberl et al., 2017; Krausmann et al., 2017; Reyna & Chester, 2014). The impact of built-environment MS therefore extends across their lifetime, dictating the flow of materials and energy for maintenance and demolition, as well as creating spatial structures that influence future urban development (Haberl et al., 2017; Krausmann et al., 2017; Pauliuk & Müller, 2014). Second, built-environment MS can be perceived as a reservoir of secondary materials which has led to concepts such as “urban mining” extending into sustainable resource management discourse (Koutamanis et al., 2018; Lanau et al., 2019). Such MS may, therefore, present opportunities for future resource efficiency such as the circular economy, which aims to maintain the value of materials and keep them in circulation within the economy and is seen as a key strategy to decouple growth from anthropogenic carbon emissions (Hart et al., 2019; Lanau & Liu, 2020). Studies have begun to address such challenges and opportunities presented by MS by quantifying the accumulation of built-environment MS within cities (Guo et al., 2020; Gutiérrez & Kahhat, 2022; Mesta et al., 2019). For example, a recent study has elaborated on the existence of lock-in effects within the city of Lima, Peru, by quantifying and mapping the MS of residential and commercial buildings (Gutiérrez & Kahhat, 2022). The study sheds light on the potential maximum growth of MS in this city and its use as a secondary resource and highlights the importance of assessing constraints relating to urban planning such as horizontal growth and the provision of basic services to better understand lock-ins.

At the forefront of such built environments, MS research is the bottom-up approach, owing to its ability to quantify and map stocks at the city- and sub-city scales, offering a high-resolution understanding as to the composition, quality, and distribution of MS while making use of context-specific data. Bottom-up material stock analysis (MSA) begins with the inventory of items, for example, m^2^ of residential buildings, which are multiplied by a material intensity coefficient (MIC), often in kg/m^2^, thus extrapolating a sample of product-level MICs over the population of corresponding product types within an area. The approach also lends itself to assessments of MS density and per capita MS accumulation that often feature in debates about the intensification of physical development of the built form globally, offering comparability between studies (e.g., Arora et al., 2019; Lanau & Liu, 2020).

Studies have also addressed the implications of urban form, often in terms of population density (Makido et al., 2012), on various indicators which have revealed, for example, that increased population density is generally associated with reduced per capita MS (Schug et al., 2022) and emissions (Gudipudi et al., 2016; Makido et al., 2012). Many now agree on the importance of urban densification and form, and thus the organization of built-environment MS, on resource efficiency (Fleischmann et al., 2021; Kennedy et al., 2015; Ramaswami et al., 2016) and the need to increase the compactness of cities within developing nations to encourage higher population densities and thus increase the utilization of built-environment MS (Creutzig et al., 2015; Huang et al., 2017; Martino et al., 2021).

The ways in which built-environment MS are provided may therefore hold important implications for resource efficiency and future urban development. As such, studies in the Global South highlight the importance of built-environment MS in sustainable urban development and often point to the need to characterize the accumulation of built-environment MS in new contexts. This is important as cities exist in various forms, from orderly street networks of orthogonal grids which are often a result of the master planning of urban areas, to more curved and organically developed areas which are often a result of more gradual local evolution (Brelsford et al., 2018, 2019). However, while there has been a recent upsurge in built-environment MS studies in the Global South (see SI section S.1), there remains limited insight into the composition and distribution of built-environment MS within cities of the Global South. This is particularly the case for India.

### Built-environment material stock accounting in India

Built-environment material stock research in India has largely focused on the material intensity of individual residential buildings (Bansal & Nandy, 2010; Bansal et al., 2014; Debnath et al., 1995; Vengala et al., 2021), with city-level studies limited to city-wide material flows (Nagpure et al., 2018) and estimations of construction and demolition waste (Ram & Kalidindi, 2017) (see SI section S.1.1). There, therefore, remains a lack of knowledge of the current composition of MS and associated challenges to resource-efficient urban development within the cities of India.

India is a key nation in the context of global sustainability given the unprecedented challenges it currently faces. India is expected to lead increases to the urban population up to the year 2050 (UN, 2018) and become the most populous country in 2023 (United Nations Department of Economic and Social Affairs, 2022). As such, it is estimated that 70%−80% of the urban infrastructure expected to exist in India by 2050 is yet to be built (IRP, 2018) with a significant demand for new buildings expected in 2030 (Ram & Kalidindi, 2017). The Government of India (GoI) has recently recommended a set of reforms to transform its urban planning capacity, noting the lack of comprehensive urban planning in the past which has resulted in significant deficits in basic service provision (India, 2021). The GoI now acknowledges the benefits of, and intends to implement, master plans in the future, seeing them as critical for managing urbanization and accommodating the basic needs of the population. However, there remains limited insight into the implications of built-environment MS composition and distribution on the resource efficiency of future urban development, particularly those resulting from the master planning of urban areas. This is particularly relevant given that the design and construction of new urban areas are required to accommodate urbanization and standards of living and that nations of the Global South (Lai et al., 2015; Long et al., 2012; Seelig, 2011) including India (Chandigarh Administration, 2022a; India, 2021) have or aim to implement master plans to construct new urban areas. However, appraisals of such master plans often focus on operational efficiencies associated with transportation and the heating and cooling of buildings (Seelig, 2011) as well as the ecological implications of urban expansion (Lai et al., 2015), thus overlooking the potentially significant resource requirements and lock-in effects of built-environment MS. It is therefore important to begin to address these research gaps to further the empirical understanding of this and the implications for future city master plans.

### Aims and objectives

In this paper, we investigate the patterns of built-environment MS accumulated in a young but rapidly developed city, which was master planned to deliver high standards of living to inhabitants via adequate access to services. Through the use of the city of Chandigarh as a topical case study, the study provides the first steps toward an improved understanding of built-environment MS within India and explores the impact of urban form on the accumulation of MS and the implications for future urban development and resource efficiency. The primary aim of the study is to understand the composition and distribution of built-environment MS within India, quantifying the MS of residential buildings and roads at the city- and sub-city-scale within India for the first time. In doing so, we also aim to provide insight into the implications for future urban master planning within India and the Global South more widely by shedding light on existing lock-in effects and opportunities for resource efficiency. Finally, we aim to ensure comparability to other studies at the product level in line with current recommendations within the literature (Schiller et al., 2018) and those assessing material intensities within India (Bansal et al., 2014; Praseeda et al., 2016; Ramesh et al., 2013; Vengala et al., 2021), with results presented and discussed in section S.2 of the SI.

## METHODS

### Case study area and scope

Chandigarh is a Union Territory and the capital city of the two northern states of Punjab and Haryana. The city was master planned to achieve high standards of living and accommodate rapid increases in the urban population in the neighboring states (see Table 1). The master plan has resulted in an orthogonal street pattern unique to cities in India and many nations of the Global South, but which has a long history as the primary strategy of urban design within cities of the United States between the 18th and early 20th centuries (Boeing, 2021; Whitehand et al., 1996). More recent master plans have adopted a grid-like urban form such as that of a small urban region in Iran which prioritizes a compact urban form to manage greenhouse gas emissions associated with built-environment stocks, for example, transport emissions and operational energy use within buildings (Seelig, 2011). Chandigarh has also achieved high standards of living, with the Census of India indicating a near-universal provision of adequate urban infrastructure and housing (Census of India, 2011). The master plan of Chandigarh has therefore resulted in a city that has a unique urban form and achieves high standards of living, which may provide valuable insight into the implications of such urban planning on the accumulation of built-environment MS. This is important given the current unprecedented rates of urbanization and demand for new urban areas to be constructed, which achieve a minimum standard of living within India and the Global South more widely.

**TABLE 1:** Key characteristics of Chandigarh’s development.

	Key facts
Development type	Chandigarh is a young and rapidly developed city and is one of the first planned cities in India (Gupta & Kavita, 2020), with construction beginning in the mid-1900s resulting from the detailed master planning of Le Corbusier (Chandigarh Administration, 2018; Rodríguez-Lora et al., 2021). A key aim of the master plan was to provide high-quality living standards for inhabitants (Chandigarh Administration, 2022b). Architectural control defines minimum requirements to ensure adequate access to piped water supply, water-borne sewage disposal, and electricity as well as safe and structurally sound housing (Sarin, 1980).
Urban form	Organized on the basis of regularly repeating neighborhood units called sectors, see supporting information Figure S.1, that are designed to be self-sufficient, with access to amenities and assets within reasonable walking distance (Chandigarh Administration, 2018) prioritized (Chandigarh Administration, 2022b). Sectors are separated by a hierarchical road network, which results in a gridiron urban form (see Figure 1), which most of the urban infrastructure, for example, water supply, follows (Chandigarh Administration, 2018). Sectors are typically 800 × 1200 m (Chandigarh Administration, 2022b) and combine to form wards, the lowest administrative division within urban areas (Census of India, 2011) (see Figure 1). Sectors are constructed in two key phases: the first phase containing 30 low-density housing sectors and the second phase containing 17 higher-density housing sectors, which were required to accommodate significant increases in the urban residential population (Chandigarh Administration, 2018, 2022b).
Residential buildings	High-rise buildings are excluded from the master plan. Stringent architectural controls have dictated the composition of housing resulting in a residential building stock that is homogeneous in its construction type (Chandigarh Administration, 2022b) (see supporting information Figure S.2). Constructed on plots allocated based on the size, that is, total square feet of the residential plot, or government housing scheme, that is, housing targeted at specific income groups to ensure affordability for poorer households (Chandigarh Administration, 2018, 2022b; Sarin, 1980).
Roads	The road network is a key element of the master plan and is designed to enable efficient traffic circulation within and between sectors (Chandigarh Administration, 2018, 2022b; Sarin, 1980). Seven road types exist based on their function, ranging from fast vehicular travel to pedestrian-only, which provide the basis for the gridiron urban form (Sarin, 1980).
Population density	9408 persons/km^2^ (Census of India, 2011).
Social progress index	86.73, ranking third out of all districts within India, scoring high in housing, basic infrastructure provision, and connectivity (Kapoor & Green, 2019).

Against this backdrop, we quantify the MS of residential buildings and roads in Chandigarh to develop an improved understanding of stock accumulation at the city- and sub-city scale within India as well as the implications regarding the provision of MS for future urban master plans within India and the Global South.

### Bottom-up material stock characterization for residential buildings and roads

We adopt a bottom-up approach to MS characterization that is comparable with approaches employed by Tanikawa et al. (2015) and Lanau et al. (2019), where the total mass of in-use stocks is estimated by multiplying the MIC by the total inventory of items in the reference area and year. The item types are a result of the archetype approach which homogenizes items, that is, residential buildings and roads, by a set of characteristics, for example, building age and construction type. As a result, an MIC is calculated for each archetype. The general approach is shown formally below:

1 $${\rm{M}}{{\rm{S}}_{m,t}} = \mathop \sum \limits_i {\rm{M}}{{\rm{S}}_{m,\;i,\;t}} = \sum {\rm{IN}}{{\rm{V}}_{i,t\;}}{\rm{x}}\;{\rm{MI}}{{\rm{C}}_{m,i,t}}$$

where MS corresponds to the total mass of material or component, $m$, of type, $i$, in the reference year, $t$. The inventory of items of type, $i$, in a dimensional unit such as local administrative boundaries, as in (Kloostra et al., 2022), for the reference year, $t$, is then multiplied by the MIC, often in mass per dimensional unit such as gross floor area, to calculate the total mass of each material in each item type which is summed over the spatial unit considered. However, bottom-up approaches generally deviate to match the units of the inventory of items with the MICs. For example, studies have overcome the lack of detailed floor area data by simplifying building inventory data to match MIC calculations (García-Torres et al., 2017) as well as using a combination of data sources and indirect calculations to fill data gaps (Condeixa et al., 2017). As a result, we adjust the method for both residential buildings (see Figure 2) and roads to suit the availability of data as outlined in Sections 2.2.1 and 2.2.2 respectively, with key assumptions presented in SI sections S.2.5 and S.3.3, respectively.

We quantify the MS in roads and residential buildings for the reference year 2011. We argue in Section 2.2.1 that the number of housing plots has remained unchanged per sector since the completion of the master plan; thus, we can provide a comparison of the stock accumulation to population and area statistics available within the Census of India (Census of India, 2011) and georeferenced data (sandeepgadhwal & devdattaT, 2018). While data on road construction are limited in India, we note the importance of estimating road MS in this context and turn to various data sources to fill data gaps, outlined in Section 2.2.2. We use the Municipal Corporation and its respective administrative wards to define the city and sub-city scales respectively, thus adopting administrative scales to assess the accumulation of MS as in Kloostra et al. (2022). We include all 26 wards in the assessment of road MS, omitting a total of 9 wards from the residential building study area due to insufficient data, enabling the calculation of inventories of buildings and their respective MICs. This corresponds to a final study area for residential building MS of 71 km^2^ accommodating 553,954 urban inhabitants and accounting for approximately 72% and 58% of the study area and population of the Municipal Corporation of Chandigarh, respectively, when compared to the road MS study area (Table 2).

**TABLE 2:** Ward-level data for residential buildings and roads.

Ward no.	Population	Area (km^2^)	No. of sectors	Estimated no. of plots	Total road length (km)
1	24,686	14.9	11 (11)	3564	178
2	32,047	7.9	3 (3)	1644	99
3	21,058	3.5	3 (3)	826	90
4	25,441	3.2	3 (3)	3689	73
5	39,075	4.2	N/A (2)	N/A	4
6	27,654	1.7	N/A (no sectors)	N/A	19
7	28,972	3.8	N/A (no sectors)	N/A	20
8	39,585	3.3	3 (3)	2895	86
9	27,567	2.5	2 (2)	2937	62
10	38,088	2.2	2 (2)	2056	52
11	47,491	2.7	N/A	N/A	36
12	47,367	2.6	2 (2)	1067	69
13	56,671	3.6	N/A (3)	N/A	81
14	51,859	1.1	1 (1)	1211	29
15	30,957	3.3	3 (3)	3531	92
16	26,593	2.2	2 (2)	3202	59
17	25,215	3.2	3 (3)	3039	81
18	30,964	3.3	3 (3)	3798	79
19	33,859	4.6	1 (2)	453	78
20	39,389	7.0	1 (1)	2522	117
21	29,654	2.1	2 (2)	2173	55
22	29,625	4.5	3 (3)	2433	113
23	74,187	3.1	N/A (no sectors)	N/A	54
24	52,070	3.5	N/A (no sectors)	N/A	50
25	45,216	2.3	N/A (no sectors)	N/A	64
26	36,297	3.0	N/A (no sectors)	N/A	45
Total	**961,587**	**99.0**	**48**	**41,040**	**1923**

Population data are retrieved from the Census of India (2011) with areas provided within georeferenced data available in GitHub (sandeepgadhwal & devdattaT, 2018). The number of sectors refers to the number of sectors for which residential building MS can be computed due to data availability, with the actual total number of sectors shown in brackets. The number of plots is estimated using the sector-wise layout plans as described in Section 2.2.1 and Figure 2. The total road length is calculated using OpenStreetMap data as outlined in Section 2.2.2.

#### Residential buildings

Architectural control drawings and layout plans provided by the Chandigarh Administration (Chandigarh Administration, 2022b) are used to calculate MICs and the inventory of items, respectively (see SI section S.2). All drawing samples contain a single three-story multi-family residential building for sectors of both the first and second phase of construction. Architectural control drawings show that each building is located on a plot type characterized by either: (1) one of the standardized plot sizes, m^2^, or (2) the plot scheme, for example, high-income group housing. The size of the plot is defined as the total footprint of the building and any outside areas such as gardens or courtyards, which is standardized through architectural control. The government housing scheme generally refers to the income band that the housing construction is reserved for, for example, low-income groups. Sector-wise layout plans locate plot types within sectors and can be used to extrapolate building sample calculations to the population of plot types (see SI section S.1). The building located on each plot is shown to be standardized through the architectural control drawings which note various standard specifications for the structural framing such as maximum building height, internal and external wall thickness, roof terrace coverage, as well as standardization of roof and floor finishes. Further, the city is primarily constructed on alluvium (Directorate of Census Operations, 2011; Kandpal et al., 2009) and is located in seismic zone IV which, following Indian design codes (BIS, 1984), controls aspects of building construction to ensure structural safety in the event of earthquakes, covering areas across multiple states of India including the large metropolitan region of Delhi (BIS, 1984). This means that the structural design of foundations and reinforced elements may be largely standardized across the city and may be similar to those housing types in such areas. To shed light on this, we compare MIC values for residential buildings within Chandigarh to those from studies across different regions of India in section S.2.3 of the SI. In total, seven plot archetypes are created, three of which relate to the government housing scheme with the remaining four relating to the standardized plot size (see Table 3). We omit wards where sufficient data are not available to describe the quantity or composition of inventory items limiting the extrapolation of MICs (see Table 2). The plot types are then related to plots shown on the sector-wise layout plans as per the master plan. A total of 48 of 56 sector layout plans are available, 13 of which provided a schedule of the number of plots for each category with a manual count required for each archetype in the remaining sectors. We use an average MIC where archetypes contain more than one drawing sample and calculate maximum and minimum errors using the maximum and minimum MIC per archetype. Where building samples for plot types are unavailable, we classify the plot by the closest available plot size for the available building samples. Where plot types within sectors are unknown, we calculate a construction-phase-specific archetype, for example, buildings constructed in phase 1 or phase 2, to homogenize buildings where plot type data are unavailable (see Table 2 and Figure 2). While we use master plan documents dating from 1957 to 2005, records demonstrate that the master plan of Chandigarh has been implemented over multiple decades (Chandigarh Administration, 2022a), with no changes to the boundaries of the district and with continued urban growth experienced to the periphery of the city (Directorate of Census Operations, 2011; Sarin, 1980). Given that sectors are of fixed size and bound by the road network, with little room for densification as per the layout plans, we assume that the total number of plots has remained unchanged since completion. Further key assumptions are presented in section S.2.5 of the SI.

**TABLE 3:** Note that the material intensity coefficient (MIC) used to calculate the total material stocks (MS) of residential buildings is taken as the product of the MIC, kg/m^2^, and the total gross floor area, m^2^, for each plot type.

Residential buildings		MIC (kg/m^2^)					
Plot type/scheme^a^	Gross floor area (m^2^)	Concrete	Brick	Steel	Clay tiles	Bitumen	Total
5 marla	212	848	866	25	104	21	1864
7.5 marla	230	848	779	25	105	22	1780
10 marla	377	838	1,135	24	110	28	2134
20 marla	656	756	923	22	98	14	1814
High-income group	253	836	1117	23	120	41	2137
Middle-income group	356	674	1048	19	107	25	1873
Low-income group	141	1022	1218	28	134	58	2459
Construction phase 1	391	872	1092	25	114	33	2136
Construction phase 2	239	846	1006	24	114	33	2023

Roads			MIC (kg/m)					
			Surface layer			Base layer		
Road type	Width, m	Recommended lane widths for roadsb (m)	Stone	Sand	Asphalt	Stone	Sand	Asphalt
Urban expressway	50	40–75	10,515	0	268	0	76,590	4951
Arterial and sub-arterial	30	30–60	6309	0	161	0	45,954	2971
Collector street	25	15–30	5258	0	134	0	38,295	2476
Local street	10	10–15	3312	409	0	18,000	0	0

See supporting information Table S.2–Table S.5 and Figure S.3 and S.4 for the material and embodied energy intensity of residential buildings by material and components and Table S.6 for a comparison to other studies within India. Also, see supporting information Table S.8 and Table S.9 for data relating to the material intensity and sensitivity analysis of roads respectively.

^a^Marla and kanal are units of land area used as part of the master plan. One marla is equivalent to approximately 272 ft^2^ or 25 m^2^, with one kanal equal to 20 marla.

^b^For roads on plain terrain as per Table 4.1 of the IRC Geometric Design Guidance, 2018 (IRC, 2018).

**FIGURE 1:**
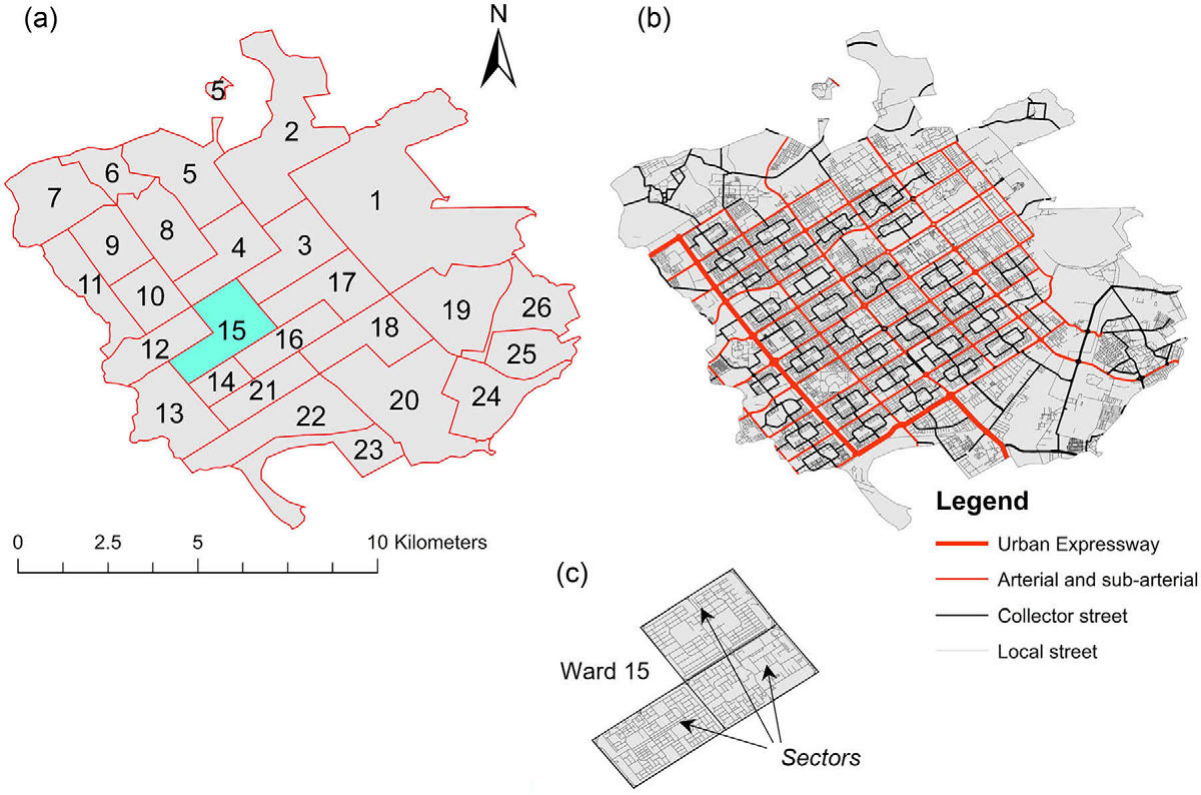
Map of the Municipal Corporation of Chandigarh as per the available georeferenced boundary data (sandeepgadhwal & devdattaT, 2018) showing: (a) the administrative wards, (b) the road network as per the road archetypes, and (c) an example ward illustrating its composition of a number of sectors. Table 1 indicates the total study area and population for both stock types based on the data available. See Supporting Information section S.6 Figure S.8 for a detailed map of the administrative divisions of Chandigarh as per the Census of India (2011).

The residential building stock is then calculated within each sector by relating the MIC, that is, total MS per plot type or construction phase, to the total number of plots by plot type, that is, size or scheme, or construction phase. The MICs are calculated using the material properties specific to India adopted in previous studies of residential building material use in India (Mastrucci & Rao, 2019; Rao et al., 2019) (see Table S.1). The sector-wise MS are then aggregated into their respective wards as per the ward map shown in Figure 1 (Census of India, 2011). Due to the diversity of bottom-up MSA applications and data availability in different contexts, MICs are often inconsistent making them difficult to compare (Schiller et al., 2018). This is important to consider in this context given that the approach must deviate from the standard MIC calculation approach, that is, adopting a kg/plot as opposed to a kg/m^2^ for the MIC of residential buildings. We therefore calculate MICs in terms of kg/m^2^ and MJ/m^2^ of gross floor area following the calculation procedure recommendations within literature (Schiller et al., 2018) to ensure improved comparability between bottom-up studies (see SI sections S.2.2 and S.2.3).

**FIGURE 2:**
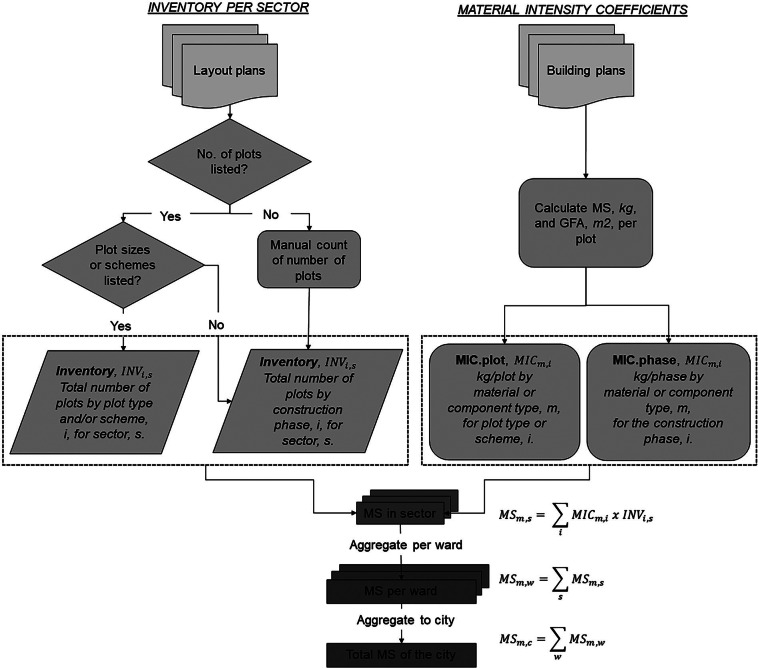
Bottom-up methodology for city and sub-city level material stocks (MS) estimation of residential buildings (authors’ own). The total MS of each material or component, *m*, for residential building archetype, *i*, in sector, *s*, are estimated by summing the product of the total number of plots per plot type, INV_*i*_, in each sector, *s*, by the material intensity coefficient (MIC) of each material or component, m, for plot type, i. The MIC refers to the total kg of material or components for each plot, with the plot defined by the plot type and/or scheme or the development phase, resulting in units of kg/plot or kg/phase, depending on the available inventory data. See Supporting Information section S.2 for a detailed description of the calculation process for residential buildings.

#### Roads

The MS of roads within the Municipal Corporation of Chandigarh and its respective wards are estimated using OpenStreetMap data to obtain the total length of different road types. We dissect road data among wards using available georeferenced ward boundary data (sandeepgadhwal & devdattaT, 2018). The study data cover all road types, excluding pedestrian and cycle paths, due to incomplete data, and therefore covers roads intended for vehicular use. We include approximately 93% of the total available raw data, equal to approximately 1923 km. The method follows the formal expression of the stock calculation as outlined in Equation (1), where the total MS of each material, $m$, for road archetype, $i$, in ward, $w$, is estimated by summing the product of the total length of each road archetype, ${\rm{IN}}{{\rm{V}}_i}$, in ward, $w$, by the MIC, kg/m, of each material, m, for road archetype, We archetype roads by comparing the function of the road as per the master plan, that is, the hierarchal road functions within Chandigarh (Chandigarh Administration, 2018), and the widths of these roads measured using Google Earth imagery, to the classifications for standard widths provided by the Indian Road Congress (IRC) (IRC, 1983), for example, expressways and arterial roads, to ensure improved comparability to stock studies in other cities such as Beijing (Guo et al., 2014) and Toronto (Kloostra et al., 2022) (see supporting information Table S.8). We therefore evaluate the MS for urban expressways, arterial and sub-arterial roads, collector streets, and local streets (see Table 3 and Figure 1). Standard specifications for the cross-sectional composition of roads are not provided within the Indian design standard publications provided by the Ministry of Urban Transport, Ministry of Road Transport and Highways, and the IRC, which generally provide information relating to road safety and quality control. We therefore turn to the assumptions made in a similar context to enable a comparison of stock accumulation between the two sectors and the city-wide composition to other cities. Studies quantifying the MS of roads in developing countries are very limited; however, MIC values from a recent study in Vietnam may offer a reasonable estimation of road MS. We adopt the MIC values estimated for roads in Vietnam to calculate road MS relating to two key road compositions of varying widths (Nguyen et al., 2019). We estimate road widths for each archetype using areal imagery from Google Earth and combine these with MIC values from Nguyen et al. (2019) to create archetype-specific MICs, kg/m. We assume the composition of roads based on road width in relation to Nguyen et al. (2019) and the visual appearance from areal images, which we verify with pothole samples within Chandigarh (Kanoungo et al., 2021). We further capture the sensitivity of the results by adopting road MIC values from an alternate study in Vietnam for which significantly lower MIC values are found (Schiller et al., 2020), discussed in Section 4 (see supporting information Table S.9).

## RESULTS

### Material stocks of residential buildings and roads in Chandigarh and its wards

**FIGURE 3:**
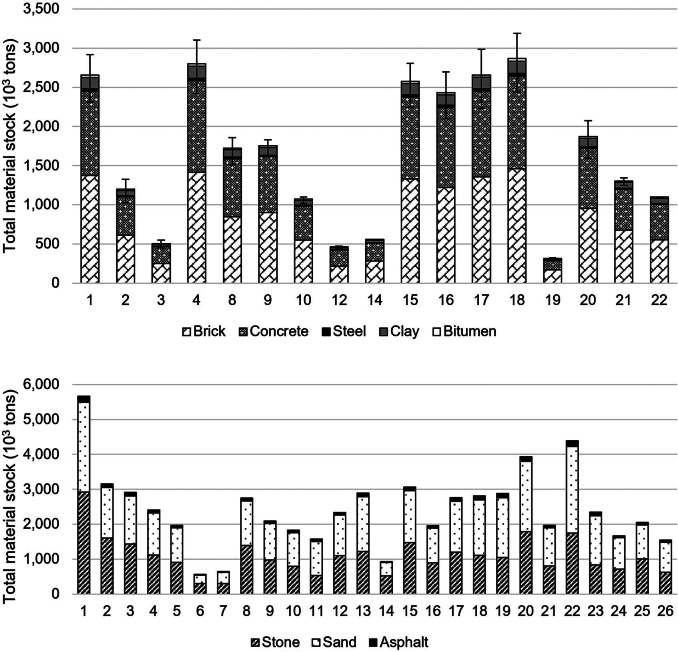
Total material stock of (top) residential buildings and (bottom) roads by material type across the wards of the Municipal Corporation of Chandigarh. See Supporting Information Table S.7 for underlying data for residential buildings and roads, respectively. Also, see Supporting Information Figure S.5 for results by residential building components.

**FIGURE 4:**
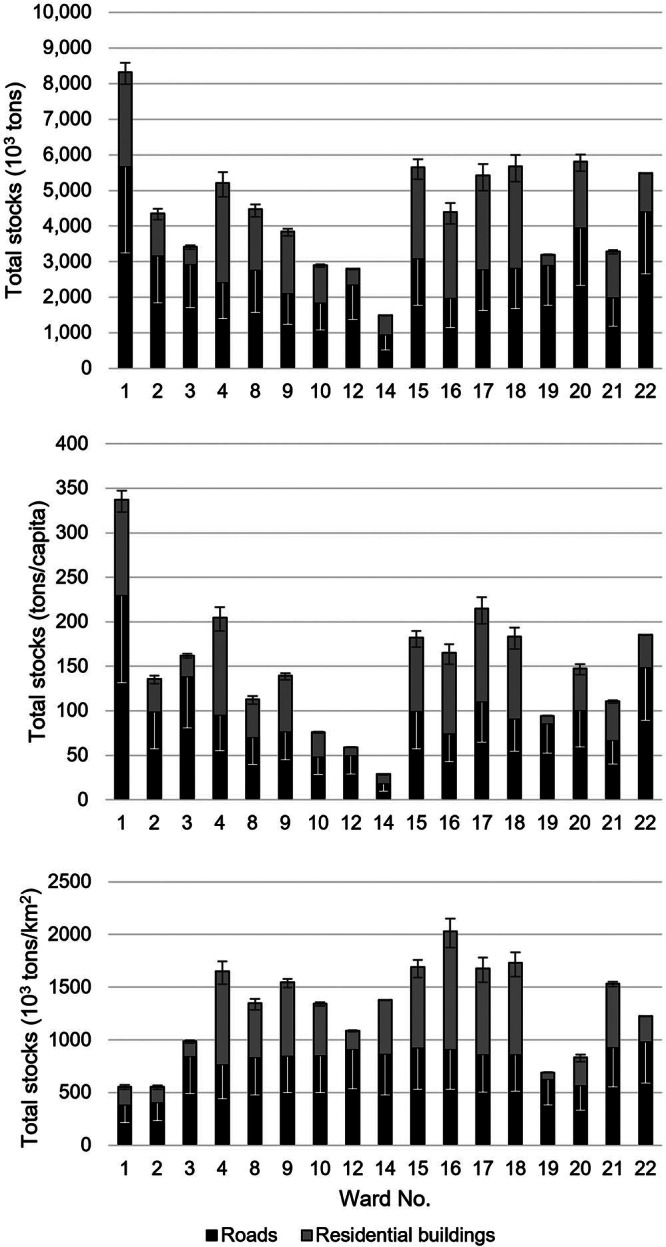
Relative composition of wards in terms of the (top) total material stock, (middle) material stock per capita, and (bottom) material stock density of residential buildings and roads. Note that error bars for residential buildings relate to maximum and minimum material intensity coefficient (MIC values per archetype, with error bars for roads relating to material stock estimations when adopting lower MIC values from an alternate study in Vietnam (Schiller et al., 2020). See Supporting Information Table S.7 for underlying data and residential building sensitivity analysis values and Supporting Information Tables S.8 and S.9 for the sensitivity analysis for roads.

We first estimate a total of 41,040 residential building plots across the 17 wards for which data are available (see Table 2). We estimate approx. 27.8 Mt of MS in residential buildings in Chandigarh, ranging in distribution from 0.3 to 2.9 Mt across different wards. Brick and concrete comprise the majority of the total MS of residential buildings and are used for the primary structural framing, accounting for 51% and 41% of the total MS, respectively. Steel reinforcement in foundations, floors, and roofs, accounts for just over 1% of the total MS with clay and bitumen used for finishes to floors and roofs accounting for the remaining 7%. The residential building stock is therefore largely composed of materials for walls (37%), floors (32%), and foundations (23%) with roof materials accounting for the remaining 9% of the total MS. The total per capita MS at the city level is found to be 50 t/cap ranging from 9 to 110 t/cap between wards. The total MS density is found to be 391 kt/km^2^ ranging from 68 to 1120 kt/km^2^. Figures 3 and 4 show the variation in stock accumulation between the wards of the Municipal Corporation of Chandigarh.

We estimate a total of approx. 63.1 Mt of MS in urban roads across the Municipal Corporation of Chandigarh ranging from 0.6 to 5.7 Mt between wards. Roads therefore account for over twice the total residential building MS. The total per capita MS of roads is found to be 66 t/cap ranging from 18 to 229 t/cap, and the total MS density is found to be 638 kt/km^2^ ranging from 168 to 981 kt/km^2^ (see Figure 4). The results highlight that the distribution of road MS is relatively uniform across the city, more so than residential building MS. In total, sand, stone, and asphalt account for 52%, 45%, and 3% of the total MS of roads with approximately half of the combined MS for roads and residential buildings used for sand in base layer construction of asphalt and concrete paved roads, that is, urban expressways and arterial and sub-arterial roads. Larger roads leading from the periphery of Chandigarh toward residential sectors, that is, urban expressways, arterial and sub-arterial roads, and collector streets, account for approximately 21% of the total road length and 39% of the total MS. Roads within sectors account for the remaining 79% of the total road length and 61% of the total MS. Thus, the results indicate that the resource requirements for roads are largely driven by the need for mobility within rather than between sectors.

**FIGURE 5:**
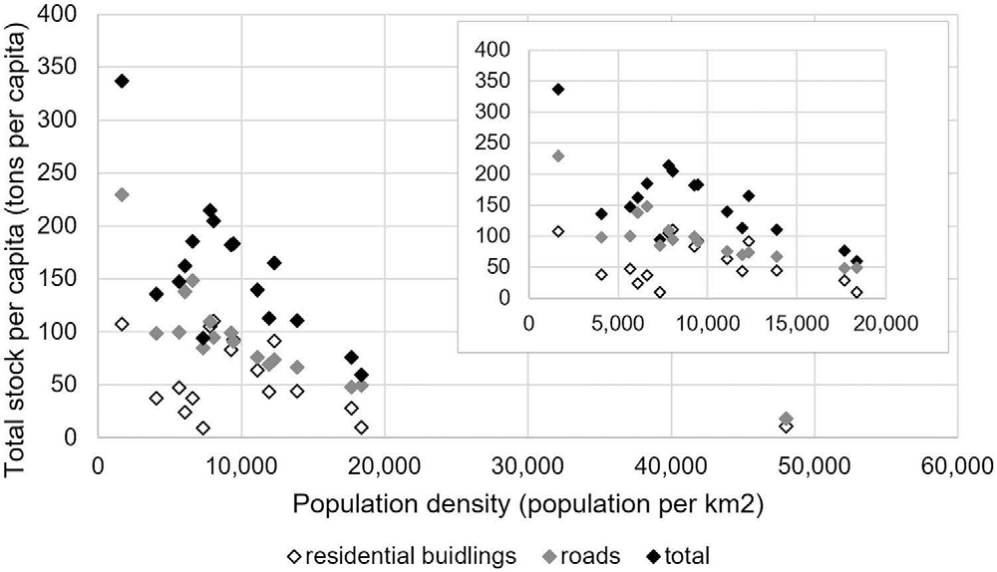
The population density versus the total stock per capita among the wards of the Municipal Corporation of Chandigarh. Note, the inset figure shows results with the maximum population density removed. Population density data for wards are calculated using the total population data available from the Census of India (2011) and the total area of wards as per the available georeferenced boundary data (sandeepgadhwal & devdattaT, 2018).

The results show that the total MS varies among wards but that the relative composition of wards in terms of road and residential building MS remains relatively uniform (see Figure 4). The variation in the available residential floor space and the uniformity of road provision among wards through a gridiron urban form of residential sectors means that the distribution of MS does not seem to be located within a central “core” as found in other cities, such as Chiclayo (Mesta et al., 2019) (see supporting information section S.5) for a brief discussion surrounding the variation in the composition of wards within Chandigarh. We also find that the accumulation of residential building and road MS is associated with population density, with results revealing that improvements to the utilization of stocks within wards, that is, lower per capita MS of both residential buildings and roads, is associated with the urban form in terms of higher population densities (see Figure 5 as well as supporting information Figure S.7 for results mapped across wards).

## DISCUSSION

### Composition and distribution of built-environment stocks in cities

#### Residential buildings

We find that the per capita accumulation of residential building MS is comparable to other cities of the Global South such as Bandung and Chiclayo and is much lower than some developed cities such as Esch-sur-Alzette and developing cities such as Rio de Janeiro (see supporting information Figure S.6 and the supporting data provided within Tables S.10 and S.11). While variability between studies in terms of the methodology, for example, the material types considered, and incoherence between the spatial resolution of MS estimations and socioeconomic indicators limits comparability, studies in Chiclayo (Mesta et al., 2019) and Rio de Janeiro (Condeixa et al., 2017) provide interesting comparative studies given their similarities and differences to Chandigarh. Chiclayo has undergone the majority of its infrastructure development and population increase in the previous four decades (Mesta et al., 2019) and exhibits a relatively widespread orthogonal street grid similar to that of Chandigarh. Further, it is largely composed of low-rise brick masonry housing with a small percentage of buildings constructed of reinforced concrete apartments. The residential building stock of Chiclayo is therefore similar to Chandigarh in terms of its homogeneity in construction type and building height. Chiclayo has a slightly larger population density and stock density while achieving a similar accumulation of MS per capita. However, the MICs, kg/m^2^, and average floor area per dwelling are found to be lower in Chiclayo than in Chandigarh. The variation in MS accumulation may therefore be explained by differences in the total household size, where larger household sizes may be experienced within Chiclayo but with a greater number of dwellings, which result in a larger stock density and population density but comparable MS per capita. This suggests that the stock of residential buildings in Chiclayo may be more compact, indicated by a larger population density of around 10,400 persons/km^2^.

On the other hand, Rio de Janeiro has a much lower accumulation of MS per capita and km^2^ than Chandigarh and Chiclayo. Despite the building archetypes in Rio de Janeiro being heterogeneous in terms of construction and building height, the city is similar to Chandigarh in that the total stock of residential buildings is mostly represented by a single building archetype. However, Rio de Janeiro is largely composed of 16-story reinforced concrete framed apartments that are between approximately 30% and 40% of the MICs calculated for Chandigarh. However, the population density of Rio de Janeiro, 5266 persons/km^2^ (Condeixa et al., 2017), is much lower than that of Chandigarh. Efficiencies associated with the utilization of MS may therefore be explained by the significantly lower MIC values resulting from higher-rise construction, with the number of stories shown to reduce MIC values in other contexts (Gontia et al., 2018; Schiller et al., 2018), including India (Bansal et al., 2014).

While we find that the per capita accumulation of residential building MS varies among wards, the Census of India shows that the distribution of household sizes remains similar (Census of India, 2011) despite uniform housing construction. As such, plot number estimations across wards may limit the accuracy of per capita stock assessments here. Such building-level and floor area information is lacking across cities within India and future work should seek to collect primary data, for example, through site surveys and remote sensing, enabling the verification of the bottom-up stock estimates here and improved comparability between bottom-up studies. This is of particular importance for an understanding of the material intensity of roads within India, which is currently significantly lacking.

#### Roads

The per capita and per km^2^ accumulation of road MS in Chandigarh is much larger than in other cities such as Beijing (Guo et al., 2014), Manchester, and Wakayama (Tanikawa & Hashimoto, 2009). Beijing has a similar MIC for expressways and arterial roads when compared to Chandigarh; however, the MIC for roads serving residential areas, which are shown to account for the majority of the road length in both Beijing and Chandigarh, is a factor of 3 larger in Chandigarh than in Beijing. Further, Chandigarh has a total road density of around 19 km/km^2^, whereas Beijing has almost half of the road density and population density of Chandigarh. This indicates that the larger MICs adopted here coupled with the greater density of roads may account for the significantly greater accumulation of road MS in Chandigarh than Beijing, despite a greater population density. However, a study of Beijing’s population within the same study year as Guo et al. (2014) highlights the significant variation in population density within the studied area of Beijing (Yao & Wang, 2014). It is therefore important to note that, while we have been able to provide a brief comparison to other cities, socioeconomic indicators and MS are often aggregated to the city level, which limits a detailed understanding of the urban development of cities. Further work addressing the sub-city accumulation of MS, particularly in relation to socioeconomic factors such as population density and standards of living, is needed to better understand the implications of urban form and human development on the accumulation of built-environment MS.

To explain the composition of Chandigarh further, and despite limited studies to draw comparison to, we compare the road-to-building MS ratio with other cities. We calculate this as the ratio of the total MS of roads to the total MS of residential buildings at the city level by aggregating MS at the ward level for which residential building and road MS estimations are available. We find this ratio to be 1.72, which is considerably larger than other studies such as the city center (0.32) and outskirts of Odense (0.86) (Lanau & Liu, 2020), Salford (0.29) (Tanikawa & Hashimoto, 2009), or Wakayama city center (0.13) (Tanikawa & Hashimoto, 2009). This ratio suggests that Chandigarh’s built-environment MS are distributed in a manner more akin to the outskirts of cities where we often find a sparser distribution of buildings with an increased length of road to connect inhabitants to services, as discussed by Lanau and Liu (2020). This finding corresponds with Chandigarh’s restriction on high-rise buildings which will inherently limit the density of urban development as floor space becomes limited and lead to a stock accumulation that is more sprawled. These results therefore highlight the need to limit urban sprawl to increase the efficiency of MS provisioning, which is in line with urban scaling observations (Bettencourt et al., 2007; Sahasranaman & Bettencourt, 2019, 2021; Zünd & Bettencourt, 2019) and general recommendations regarding the design of new urban areas (Gudipudi et al., 2016). It is important to note that the ratio accounts only for residential buildings, and it is therefore expected that the actual road-to-building stock ratio would reduce further. When adopting the alternate MIC values for roads estimated in Vietnam (Schiller et al., 2020), we find that the total MS of roads is reduced by 41%, and thus, the city-level stock ratio reduces from 1.72 to 1.01. However, this remains significantly higher than in other cities and underlines that the sprawled urban form is a key driving factor in the resulting MS accumulation.

#### Implications for future urban development and resource efficiency

The results reveal that around 67% of the total MS of roads and residential buildings is belowground, that is, it is used to construct either the base layer of roads or the foundations of residential buildings. The total underground MS of residential buildings and roads are 23% and 86%, respectively, indicating that the majority of the total MS may be considered as “lost stocks” and thus offer limited potential for secondary reuse. Road MS dominate overall MS accumulation and we find that this stock has limited potential for secondary use beyond standard construction practice. Base layer construction of roads accounts for approximately 86% of the total MS of urban roads and 60% of the total city-wide MS. Sand and stone are the primary resources for base layer construction and are limited in their recoverability. As such, the resources used for road construction offer limited opportunities for secondary use. The stock of materials in residential buildings is largely composed of brick for walls and foundations and reinforced concrete for floors, foundations, and roofs (see supporting information Figure S.5). These materials account for only 16% and 13% of the total city-wide MS, respectively; however, they may offer a greater opportunity for resource efficiency strategies. In situ concrete is generally not suitable for deconstruction given that materials are difficult to separate without damaging components and that any separated concrete components are generally downcycled and crushed (Densley Tingley, 2012). As a result, the reuse of foundations from demolished buildings may present the most appropriate strategy for secondary resource use. Given that building designs in terms of typology and material use are largely standardized, future buildings may be able to reuse foundations due to similar loading criteria. However, the lack of structurally code-compliant design in Chandigarh (Chandigarh Administration, 2022a) may impact the quality and reusability of materials and components. The master plan for Chandigarh to 2031 (Chandigarh Administration, 2022a) proposes identifying opportunities for the densification of first-phase sectors due to the lack of land availability in the city, and the provision of housing for the urban poor. One option for densification could be through the vertical extension of existing buildings. However, additional materials would likely be required to reinforce the existing structures to facilitate this, that is, strengthening of columns and foundations to accommodate increased load, and expansion may still be limited without significant increases to the height of buildings (Gillott et al., 2022). Alternatively, poor-quality housing may become a useful secondary resource, which may have the potential for deconstruction and recirculation into the economy, particularly for non-reinforced components such as brick walls, which account for approximately 11% of the total MS of Chandigarh.

The results of the study therefore indicate that the master plan has resulted in a considerable “lock-in” effect, which significantly restricts opportunities for urban development within the existing urban structure. As a result, it is reasonable to assume that future development will be accommodated by either vertical extension, where structurally feasible, or more likely, the demolition of existing buildings with excessive waste. These results highlight the importance of understanding the implications of built-environment MS on the resource efficiency of future urban development when master planning new urban areas. Future work should therefore address such MS implications within existing master plans in other contexts, such as that of a small urban region in Iran (Seelig, 2011). It is also important for future work to address the relationship between the accumulation of such stocks and the levels of development at sub-city scales, particularly in terms of access to basic services, to improve comparability between studies and better elaborate resource-efficiency strategies aiming to decouple human development from environmental impact within the built environment.

## CONCLUDING REMARKS

To conclude, we show here that the unique urban planning case of Chandigarh, resulting from the master planning of the city centered around universally high living standards, has driven a significantly larger ratio of road-to-building MS than the center and outskirts of other cities. While residential building MS are comparable to other cities in the Global South, road MS have accumulated to a much greater extent than some rapidly urbanizing and developed cities. Considerations of connectivity and living standards within and across low-rise residential areas have resulted in an urban form that presents “lock-ins” limiting future urban development without demolition of existing stock. However, the existing stock was not considered for use as a secondary resource, and it is now important to understand demolition waste flows and their potential for recirculation into the economy. This is particularly important due to the significant amount of waste expected to be generated in India from the construction sector due to rapid urbanization (Ram & Kalidindi, 2017) and the planned redevelopment of Chandigarh to 2031 (Chandigarh Administration, 2022a). We therefore provide empirical evidence pointing toward the need to integrate material stock thinking into urban planning and development, with a particular focus on transport infrastructure and connectivity. Future integration of this within assessments of living standards will be valuable to enable an empirical understanding of the relationship between built-environment MS and the development outcomes of inhabitants within cities.

## Supplementary Information

**Supporting Information S1**: The Supporting Information provides additional information regarding existing literature, the case study city and data available as well as the data used to compare the accumulation of MS in other cities. It also contains a comparison of the material intensity values estimated here to those in existing studies within India. Section 1 provides a brief review of existing literature within the Global South and India specifically. Section 2 provides an overview of the data and assumptions for the material stock estimation of residential buildings as well as a comparison of the MIC values to those in India based on the location and building archetype. Section 3 provides an overview of the data and key assumptions for material stock estimations of urban roads as well as the results of the sensitivity analysis. Section 4 compares the city-level material stock results to other cities with section 5 briefly discussing the sub-city variation in material stock accumulation. Finally, a detailed map of Chandigarh as per the census is provided in section 6.

## Data Availability

The data that support the findings of this study are available from the corresponding author upon reasonable request.
