# Supplementary material for: Built-environment stocks in the context of a master-planned city: A case study of Chandigarh, India
Source: J Ind Ecol. 2024 Apr 5;28(3):573–88. doi: 10.1111/jiec.13466 (PMC13083536; doi:10.1111/jiec.13466)
Supplement: Supplementary file 1 — Supporting Information S1: The Supporting Information provides additional information regarding existing literature, the case study city and data available as well as the data used to compare the accumulation of MS in other cities. It also contains a comparison of the material intensity values estimated here to those in existing studies within India. Section 1 provides a brief review of existing literature within the Global South and India specifically. Section 2 provides an overview of the data and assumptions for the material stock estimation of residential buildings as well as a comparison of the MIC values to those in India based on the location and building archetype. Section 3 provides an overview of the data and key assumptions for material stock estimations of urban roads as well as the results of the sensitivity analysis. Section 4 compares the city-level material stock results to other cities with section 5 briefly discussing the sub-city variation in material stock accumulation. Finally, a detailed map of Chandigarh as per the census is provided in section 6. [file 44498_2024_2803015_MOESM1_ESM.docx]

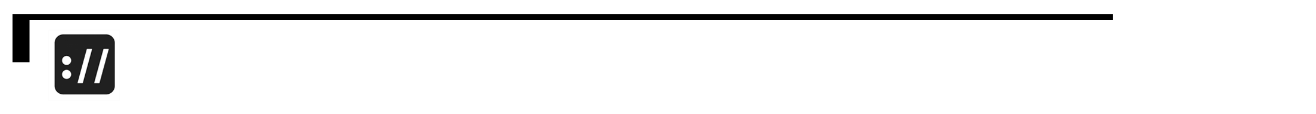


# SUPPORTING INFORMATION FOR:

Mihkelson, W., Arbabi, H., Hincks, S., & Densley Tingley, D. (2024.)

Built environment material stocks in the context of a master planned city: A case study of Chandigarh, India. *Journal of Industrial Ecology.*


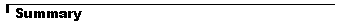


The Supporting Information provides additional information regarding existing literature, the case study city and data available as well as the data used to compare the accumulation of MS in other cities. It also contains a comparison of the material intensity values estimated here to those in existing studies within India. Section 1 provides a brief review of existing literature within the Global South and India specifically. Section 2 provides an overview of the data and assumptions for the material stock estimation of residential buildings as well as a comparison of the MIC values to those in India based on the location and building archetype. Section 3 provides an overview of the data and key assumptions for material stock estimations of urban roads as well as the results of the sensitivity analysis. Section 4 compares the city-level material stock results to other cities with section 5 briefly discussing the sub-city variation in material stock accumulation. Finally, a detailed map of Chandigarh as per the census is provided in section 6.


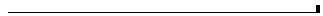


## Introduction

While studies quantifying built environment MS have increased in the past decade, much of the research quantifying built-environment MS accumulation focusses at national levels (see Lanau et al., (2019)) quantifying the MS of residential (Bergsdal, Brattebø, Bohne, & Müller, 2007; Ortlepp, Gruhler, & Schiller, 2018; Wiedenhofer, Steinberger, Eisenmenger, & Haas, 2015) and non-residential (Ortlepp, Gruhler, & Schiller, 2015) buildings as well as transport infrastructure (Miatto, Schandl, Wiedenhofer, Krausmann, & Tanikawa, 2017; Tanikawa, Fishman, Okuoka, & Sugimoto, 2015; Wiedenhofer et al., 2015). City-level assessments in the Global South are limited and often concentrated in China due to the availability of data (J. Guo et al., 2020; Z. Guo, Hu, Zhang, Huang, & Xiao, 2014; Hu et al., 2010; Huang, Han, & Chen, 2017; Mao, Bao, Huang, Liu, & Liu, 2020), however studies are emerging in other countries such as Peru (García-Torres, Kahhat, & Santa-Cruz, 2017; Mesta, Kahhat, & Santa-Cruz, 2019), Indonesia (Surahman, Higashi, & Kubota, 2017) and Brazil (Condeixa, Haddad, & Boer, 2017). Studies have now quantified the stock of residential buildings (García-Torres et al., 2017; Z. Guo et al., 2014; Mesta et al., 2019), non-residential buildings (Huang et al., 2017; Mao et al., 2020; Tanikawa & Hashimoto, 2009), and roads (Z. Guo et al., 2014; Huang et al., 2017; Mao et al., 2020) and demonstrated an ability to map these results at high resolution and over large temporal scales. For example, a comprehensive study assessing over one-hundred years of MS accumulation has revealed the dynamics of development at the sub-urban scale and highlighted the need to account for environmental impacts and waste generation into future plans for urban redevelopment (J. Guo et al., 2020). The sectoral transformation of this area from industrial to predominantly residential highlights the implications of lock-in effects which limit future urban development and require extensive demolition activities to renew urban areas. It also further highlights the need to understand MS at the product-level to better inform resource efficiency strategies such as urban mining or building reuse to ensure that lock-in effects minimize waste. However, there remains significantly limited insight into the composition of built environment MS accumulated within Indian cities and the associated implications for resource efficiency and urban development. Further, there remains a clear gap in current research addressing the accumulation of built environment MS within urban areas master planned to accommodate urbanization and achieve high standards of living in the Global South. Such an understanding is important given the unprecedented demand for new urban areas given the expected rates of urbanization in many cities of the Global South. As outlined in the main text, this is particularly important for the nation of India.

### Material stock accounting in India

Built-environment stock research in India has, to date, largely focused on material and embodied energy (EE) intensities of individual residential buildings (Bansal & Nandy, 2010; Bansal, Singh, & Sawhney, 2014; Debnath, Singh, & Singh, 1995; Vengala, Ramesh, Dharek, Krishna, & Kumar, 2021). The EE relates to the total energy required to produce and transport materials as well as the energy required to construct the product, e.g., building, road etc., (Praseeda, Reddy, & Mani, 2016) and is often reported in studies focused on built-environment material use in India. Studies have evaluated the potential for energy efficiency in buildings, focusing on construction material use (Bansal et al., 2014; Mastrucci & Rao, 2019) and operational energy demand (Mastrucci & Rao, 2019). Bottom-up approaches have been used to estimate the resource requirements needed to provide minimum standards of living nationally (Mastrucci & Rao, 2019; Rao, Min, & Mastrucci, 2019). This has been combined with district-level statistics to estimate the material implications of closing deficits in living standards through the assessment of city-wide cement material demand for Delhi and Chandigarh (Nagpure, Reiner, & Ramaswami, 2018). The bottom-up approach has also been applied nationally to estimate the energy requirements needed to meet basic standards of living through the provision of adequate infrastructure (Rao et al., 2019) and to meet housing demands (Mastrucci & Rao, 2019). However, national estimates fall short of offering insight into material efficiency strategies at the material- and product-level, as well as an understanding of the intensity of the built-form within cities. City-level studies in India are therefore limited to city-wide material flows (Nagpure et al., 2018) and estimations of construction and demolition waste (Ram & Kalidindi, 2017).

## Residential buildings

The uniform composition of housing within Chandigarh is evident through the building samples and documentation provided by the Chandigarh Administration and the Directorate of Census Operation in Chandigarh (Chandigarh Administration, 2022c, 2022b; Directorate of Census Operations, 2011). The uniform typology of buildings of Chandigarh, i.e., three-story residential buildings constructed using the same structural framing and materials, is dictated by the architectural control due to the master planning of the city and means that a single city-level archetype is appropriate for comparison to other studies of building archetypes. However, due to data provided by the Chandigarh Administration (Chandigarh Administration, 2022c) we are able to further the archetype classification to create Chandigarh-specific archetypes. As a result, we can more accurately map the material intensity coefficient (MIC) data to the inventory of items within each sector to calculate the total MS, as presented in the main text. Building sample drawings can be found following the link to the architectural control drawing repository on the Chandigarh Administration website within the citizen facilitation section (Chandigarh Administration, 2022a).

### Inventory of items

In the main text we outline the process and data used to create the inventory of items for Chandigarh-specific archetype classifications, i.e., where we further homogenize building samples by plot type and government housing scheme. The inventory of items, i.e., the number of different plot types or government housing schemes within a sector, are collected from layout plans. Figure S.1 shows an example layout plan for sector 35. The inventory of items at the sector level are then aggregated to their respective wards as per the Census ward map (Census of India, 2011), see Figure 1 of the main text, and combined with the MIC values outlined in the following section. Inventory data pertaining to plot types and numbers within sectors can be found on the Chandigarh Administration website within the citizen facilitation section (Chandigarh Administration, 2022e) or alternatively on the website for the Department of Urban Planning Chandigarh (Chandigarh Administration, 2022d).


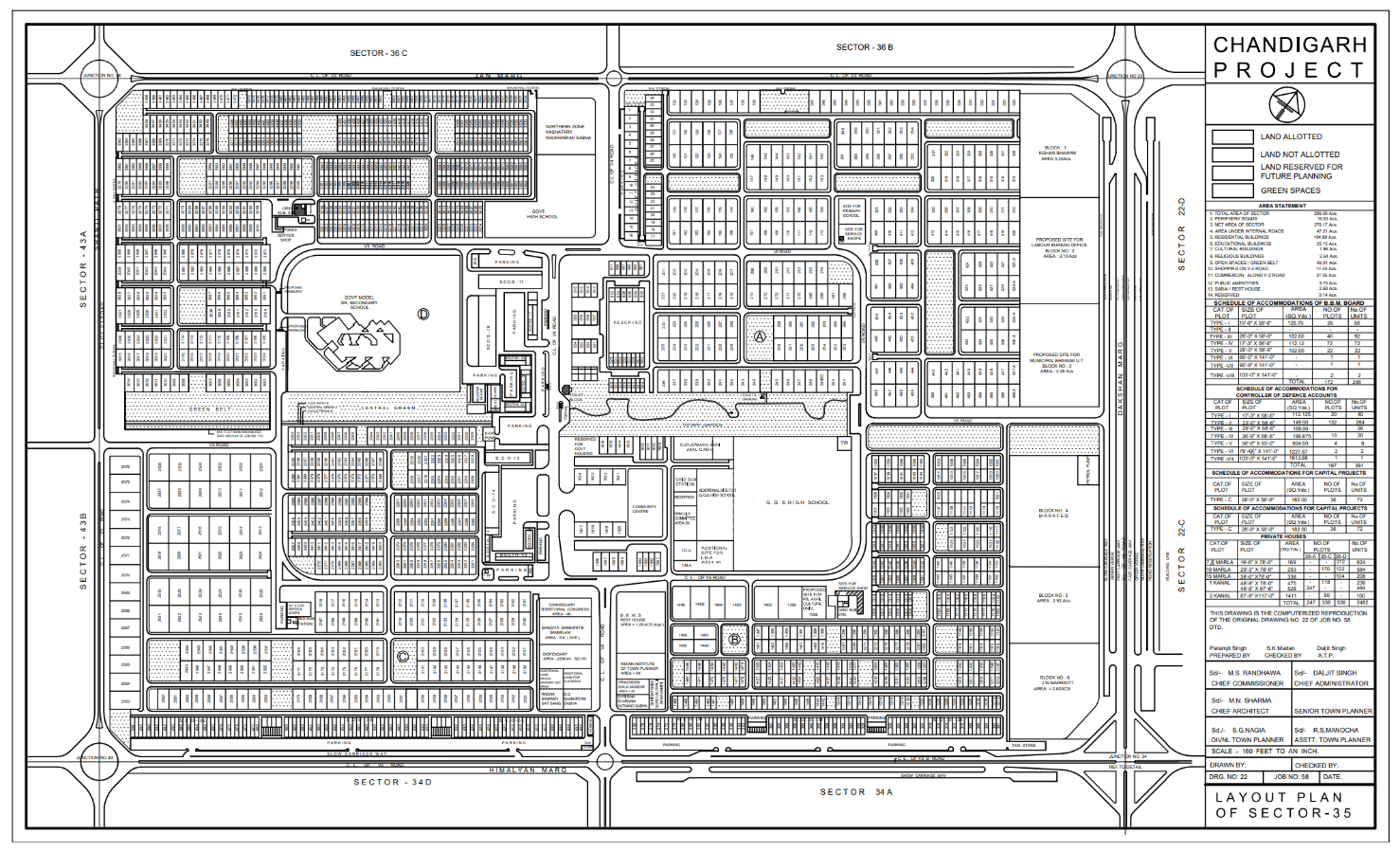


Figure S.1: Example layout plan used to calculate the inventory of items at the sector-level. Each grid, e.g., a, b, c, and d, within sectors corresponds to a sub-sector, summing to 4 in total for each sector. The number of each plot type is summarized within the schedule of accommodation for different project types within the drawing notes. Where the schedule is not provided, and the plot types are labelled within each sub-sector, the plot types are counted manually and related to the construction phase of the sector.

### Material and embodied energy intensity coefficients

Due to the diversity of bottom-up MSA applications and data availability in different contexts, MICs are often inconsistent making them difficult to compare (Schiller et al., 2018). Our starting point is to address the calculation of MICs and embodied energy intensities (EEI) for residential buildings, the latter of which is undertaken to ensure greater comparability to existing studies within India, see section S.2.3. We therefore adopt EE values used by Mastrucci & Rao, (2019) to calculate residential building EE in India in an attempt to reflect the prevailing material production and construction practices of India, see Table S.1. From here we use the same building samples to create building archetypes specific to Chandigarh with which to further improve the accuracy of MS estimations, as presented in the main text.

Building samples are collected through the Chandigarh Administration Citizens Facilitation which stores architectural control drawings for various plot types and government housing schemes. We use as many drawings as are available relating to the inventory of items found through the layout plans. In total, 12 building samples are collected for superstructure MIC calculation and 2 building samples collected from the Chandigarh Housing Board (Chandigarh Housing Board, 2022) to calculate substructure MICs due to the greater detail of foundations provided in comparison with architectural control drawings. A typical architectural control drawing is shown in Figure S.3. Table S.1 contains the material property data used to calculate the total mass and embodied energy of materials and components for all building samples.

The MIC for comparison to other studies is calculated as the total mass of material or component type for all building samples which is summed to arrive at a total kg of material or component. From here the total gross floor area (GFA), as defined in (Schiller et al., 2018), is calculated for each building and summed to result in the total floor area of the building archetype. Finally, the MIC is calculated by dividing the total MS by the GFA. The EEI is calculated by multiplying the mass of material, *kg,* by the EE coefficient, *MJ/kg*, using India specific EE values (Mastrucci & Rao, 2019). Table S.2 and Table S.3 provide a summary of the material and EEI of city-level and Chandigarh-specific archetypes for multi-family, 3 story residential buildings (MFH-3F) by material. Table S.4 and Table S.5 provide the same information but disaggregated by building component.


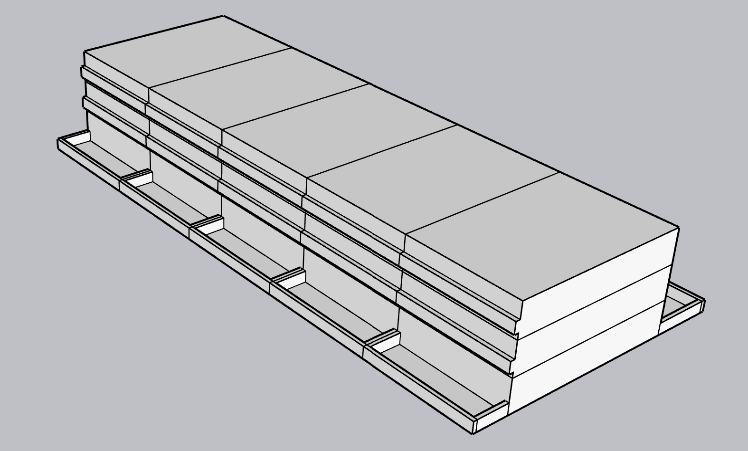

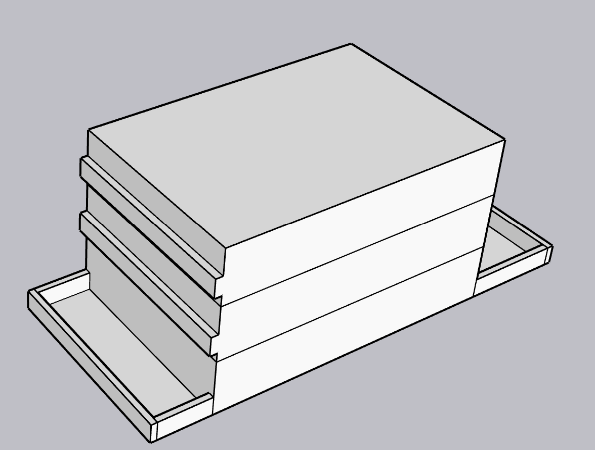

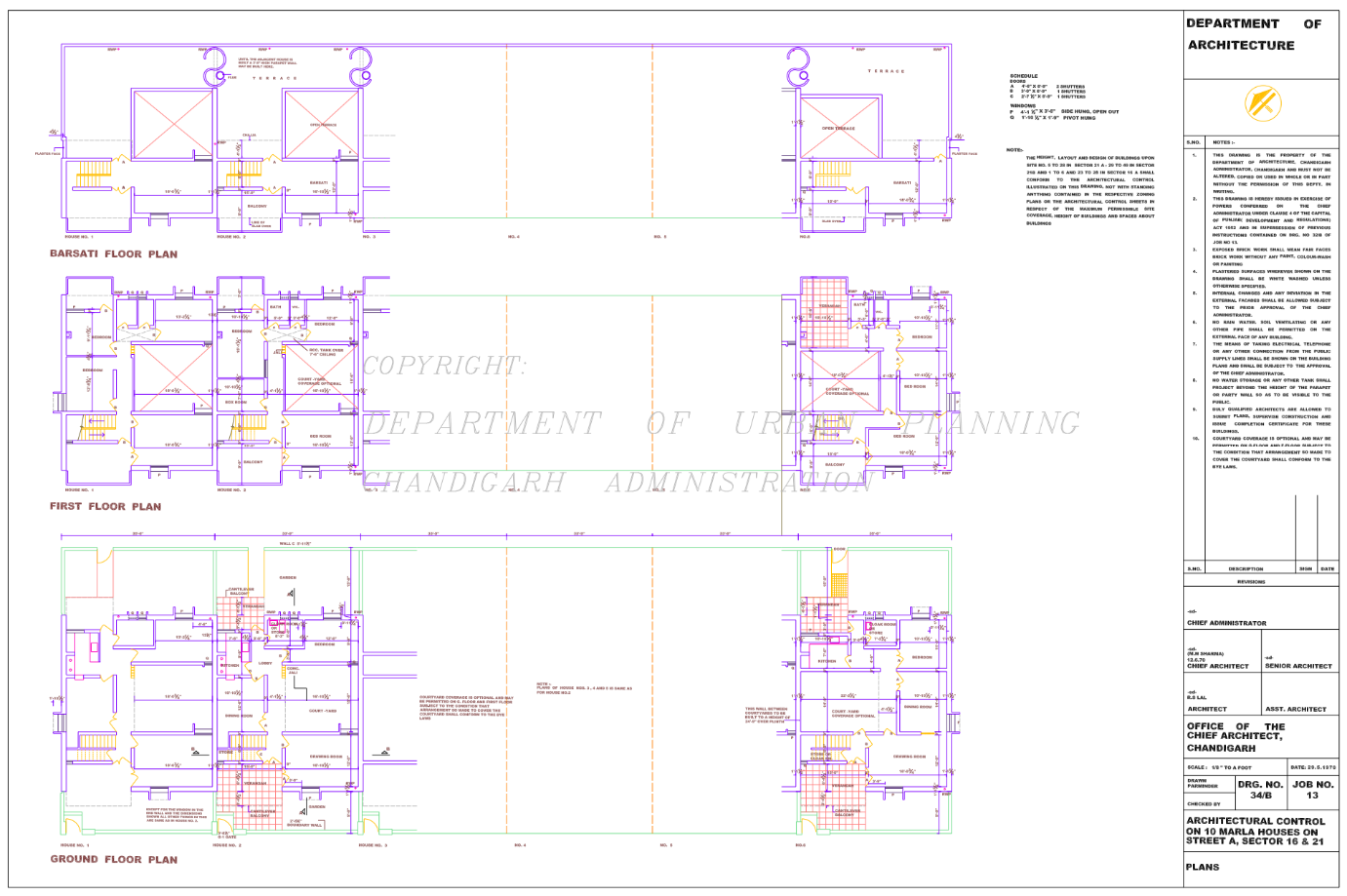


**A**

**B**

**C**

Figure S.2: (A) Example architectural control drawing showing the plan view for a 10 marla housing plot type (Chandigarh Administration, 2022a). (B) Simple 3D model of a row of 10 marla housing (authors own) and (C) a simple 3D model of a single 10 marla plot (authors own), where the plot size includes the footprint of the building and the two external areas.

Table S.1: Material properties used to calculate the total mass and embodied energy of residential buildings, raw values are provided by (Mastrucci & Rao, 2019)

| **Material** | **Density (kg/m^3^)** | **Embodied energy (GJ/unit)** | **EC (kgCO2/ton)** |
| --- | --- | --- | --- |
| Reinforced concrete slab (1% steel) | 2300 | 3.65 (m^3^) | 0.067 |
| Reinforced concrete foundation (0.5% steel) | 2300 | 2.56 (m^3^) | 0.122 |
| Brick | 1800 | 2.41 (m^3^) | 1.82 |
| Steel | 7800 | 20.62 (ton) | 2.98 |
| Clay tiles | 2300 | 3.33 (ton) | 0.334 |
| Bitumen membrane | 1100 | 2.98 (ton) | 0.334 |

Table S.2: Data_from_table_3_in_manuscript and Figure S.4 in SI. Material intensity for residential building archetypes and the city-level archetype (MFH-3F) by material.

Table S.3: Data from Figure S.4 in SI. Embodied energy intensity for residential building archetypes and the city-level archetype (MFH-3F) by material.

Table S.4: Material intensity for residential building archetypes and the city-level archetype (MFH-3F) by component.

Table S.5: Embodied energy intensity for residential building archetypes and the city-level archetype (MFH-3F) by component.

### Material intensity of residential buildings in comparison with other studies

Here we present results for the MIC and EEI for the city-level archetype of residential buildings which are calculated to provide comparability with other product-level and bottom-up studies, as outlined in section 2.2 of the main text. Residential buildings in Chandigarh pertain to a single archetype containing the same structural framing, number of stories and material specifications and vary only in the GFA provided. The MIC and EEI for MFH-3F in Chandigarh is found to be 2,550 kg/m^2^ and 4,190 MJ/m^2^. Table S.2 of the main text shows that there is little variation in MIC and EEI across buildings samples. Thus, we find that the architectural control of Chandigarh has resulted in a population of residential buildings that require broadly similar quantities of material and EE per GFA, with variations largely captured by differences in brick and concrete consumption, see Table S.2 - Table S.5.

We find the EEI for residential buildings in Chandigarh is very similar to other studies in India considering similar framing and story height. These studies are compared in Table S.7, with results suggesting that seismic and climatic zone may enable broader archetype classification in the data constrained context of India. It is expected that the climatic zone will not have a significant effect on the embodied impact of buildings as operational or mechanical design may be standardized by climatic zone to ensure operational efficiency within the environment as opposed to adding significantly more material to the building. On the other hand, the seismic zone is used to standardize the specification and detailing of some structural elements to ensure safety during seismic activity (Kisan, Sangathan, & Nehru, 1993). As such, we are likely to see similarities between these regions for code compliant structures which may aid in the estimation of residential building MS where data is severely lacking. For example, the values for Chandigarh and Delhi are found to be almost identical, with both situated within seismic zone IV and in composite climates. However, we see a range of values for those building samples in seismic zone III and future work should seek to understand the similarities and differences in embodied impacts of residential buildings per seismic and climatic zone. The EEI found is within the range of 3,000-5,000MJ/m^2^ for residential buildings in India (Debnath et al., 1995), however we find differences among other studies considering a similar archetype classification of residential building in India. Praseeda et al., (2016) find values ranging between 3,790-4,250MJ/m^2^ across a large range of built-up building areas, m^2^, with Bansal et al., (2014) finding values of 3,536MJ/m^2^ and 3,382MJ/m^2^ for three- and four-story residential buildings within the same climatic and seismic zones. Vengala et al., (2021) find a value for four-story residential buildings in Vijayawada, a city in the south-east of India in different climatic and seismic zones to Chandigarh, of approximately 3,100MJ/m^2^.

While we are able to provide a brief comparison of EEI values in India here, the lack of transparency in material calculations and standardized units of EE limit the comparability of studies and should be considered in future work comparing product-level material and energy consumption.


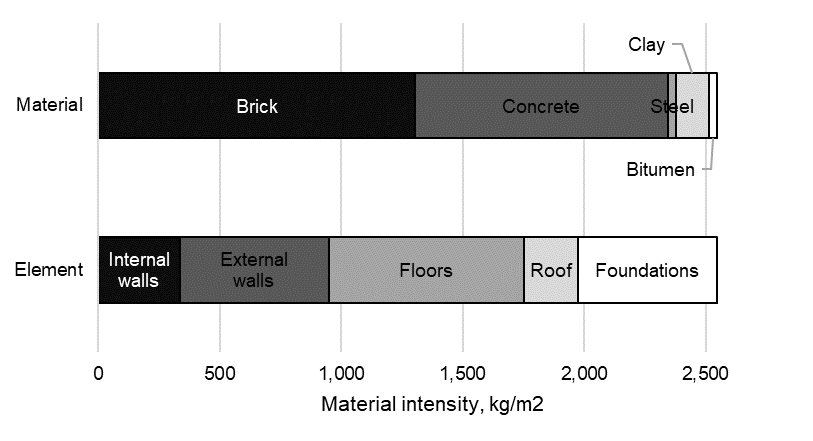

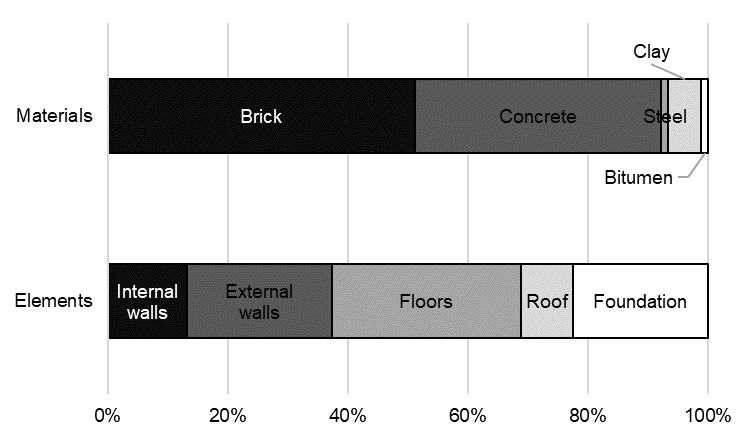


Figure S.3: Material intensity, kg/m^2^, for the city-level archetype of residential buildings in terms of the relative (top) and absolute (bottom) MIC by material and component.


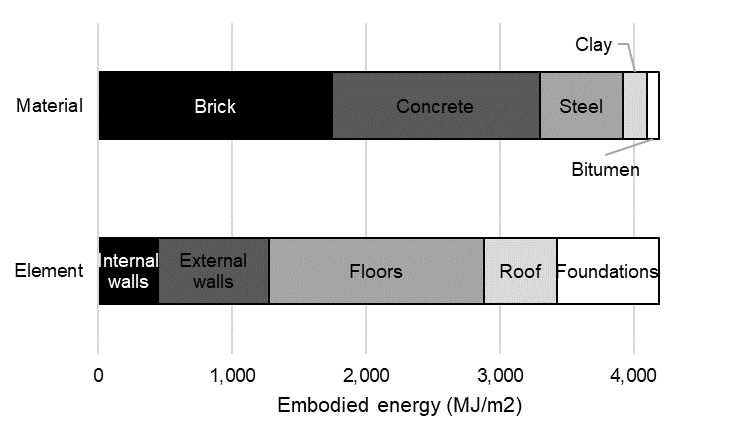

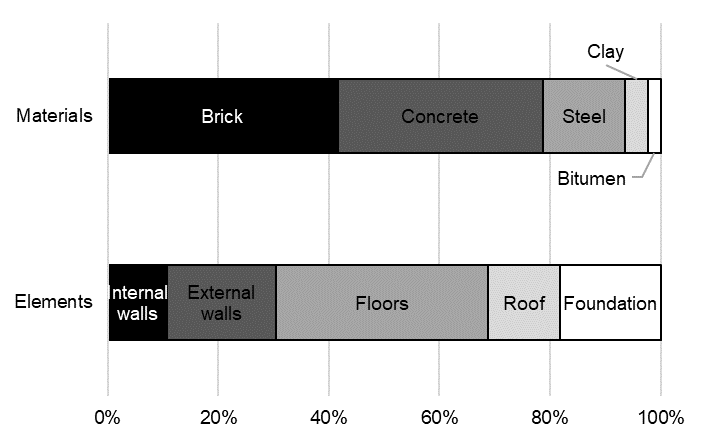


Figure S.4: Embodied energy intensity, MJ/m^2^, for the city-level archetype of residential buildings in terms of the relative (top) and absolute (bottom) EEI by material and component.

Table S.6:Comparison of embodied energy intensity (MJ/m^2^.GFA) for residential buildings in India based on the city and seismic and climatic zone. Archetypes from other studies are selected which most closely relate to the composition of building archetypes within Chandigarh.

| **Study** | **City** | **Seismic zone** | **Climatic zone** | **Archetype** | **EE (MJ/m2)** |
| --- | --- | --- | --- | --- | --- |
|  | Chandigarh | IV | Composite | 3-story, MFH, brick masonry envelope and RC structural frame | 3,460 |
| (Bansal et al., 2014) | Delhi | IV | Composite | 3-story, burnt clay brick masonry-based house | 3,564 |
| (Ramesh, Prakash, & Kumar Shukla, 2013) | Allahabad | III | Composite | 4-story, MFH, brick envelope and RC structural frame | 7,350 |
| (Praseeda et al., 2016 | N/A | N/A | Moderate | 4-story, Rubble stone masonry foundation, RC frame structure with burnt brick masonry infill, RC roof and ceramic, mosaic tile flooring | 3,790 |
| (Praseeda et al., 2016) | N/A | N/A | Composite | 1-story, Rubble stone masonry foundation, load bearing stone masonry structure, RC roof and cement concrete flooring and Rubble stone masonry foundation, RC frame structure with burnt clay brick masonry infill, RC roof and mosaic tile flooring respectively | 1,010- 4,120 |
| (Vengala et al., 2021) | Vijayawada | III | Warm and humid | 3-story, RC framed structure with brickwork in sub- and super-structure | 3,100 |

### Material stock of residential buildings

In the main text we outline the total stock of material in residential buildings by material for each ward. Figure **S**.5 shows the same information disaggregated by building component.

Figure S.5: Total material stock by component across the wards of the Municipal Corporation of Chandigarh.

Table S.7: Data_from_figures_3_and_4_in_manuscript. Data underlying ward- and city-level MS results.

### Summary of key assumptions

While architectural control drawings show standard dimensions for the structural framing and floor and roof finishes, a number of assumptions are made to fill gaps in data per drawing and layout plan. We assume standard dimensions and detailing of buildings where data is not available as a result of the stringent architectural control. Architectural control drawings generally show the roof as half thickness of the floors. The majority of drawings show the roof thickness, all of which coherently show a 4.5” RC slab with 1.5” clay tiles and 2 layers of bitumen. Where these details are not provided, we assume these specifications in line with the architectural control. Where the thickness of concrete is not specified for roofs, we calculate the thickness by the difference between the overall specified thickness and roof finishes as per the assumptions or the specifications on drawings. The thickness of bitumen is not specified explicitly and we therefore assume the thickness is 5mm as per (Mastrucci & Rao, 2019). Most drawings show floors with 1.5” ceramic tiles for finishes and we assume this specification in line with the architectural control where details are not provided. Structural drawings specifying foundation details show that foundation thicknesses are related to the thickness of the wall. Walls are shown to be either 9” (internal walls) or 13.5” (external walls). Thus, we assume foundation (strip footings) correspond to the wall thickness for all architectural control drawings. As discussed in the main body, the ground conditions of Chandigarh are uniform and the building typologies homogeneous, thus we assume that foundation design is the same across buildings (strip footings under load bearing walls constructed of concrete, RC and brick). Most drawings specify wall thicknesses for internal and external walls of 9” and 13.5” respectively. We assume this thickness for drawings where these are not specified. Brick jails (walls surrounding the roof terrace area) are constructed of brick of half story height and are therefore considered as external walls. For the 5 marla plot type (126m^2^), the width of the building is not given. We assume the width is the same as the 7.5 marla plot and verify the overall plot size (130m^2^). Plot types often correspond to particular sectors which belong to their own construction phase as per the masterplan. We therefore assume this architectural control is generalized as per the phase and create two building archetypes for phase 1 and 2 to account for unknown plot types within sectors. We assume that steel accounts for 1% of the total volume of concrete for which is used for reinforcing as per the data available from (Mastrucci & Rao, 2019). This value corresponds to reinforced slabs which are used for the floor system of all buildings. The drawings show no reinforcement in beams or columns due to load-bearing brick and masonry walls on the interior and exterior of the building and reinforced slabs used for floor systems with no structural beams specified. We assume that construction methods leading to 2011 are the same as in the masterplan, with drawings showing no difference between original drawings (dating from 1956) to revised drawings (dating up to 2008).

## Urban roads

### Inventory of items

We use Geofabrik (<https://download.geofabrik.de/asia/india.html>) to obtain transport infrastructure data from openstreetmap (OSM). The road types are split into various categories such as residential, path, primary, and secondary etc. and are highlighted on the respective maps of Chandigarh in ArcMAP below. Residential categorizations of roads are most common, accounting for 54% of the total length of roads downloaded. We evaluate primary, residential, secondary, service, tertiary, and trunk road types including their respective *link* data where necessary (as these contain details such as roundabouts and are merged into a single road type). We therefore account for ~93% of the total length of road data available, omitting roads that are unclassified as well as those limited in coverage and thought to be unrepresentative of the total stock (e.g., cycleways and pedestrian/paths).

### Material intensity coefficients

As outlined in the main text, there is limited information relating to the composition of roads in India. We therefore combine various data sources to archetype roads by width, function and construction type, see Table S.8, and assess the sensitivity of the results to the MIC formulation by calculating MICs based on significantly lower kg/m^2^ values for roads in an alternate study of roads in Vietnam, see Table S.9.

Table S.8: Road archetypes relating Chandigarh's road system with recommendations as per the Indian Road Congress (IRC, 2018), approximated road widths using Google Earth Imagery, and road compositions and MIC as per the study of road stocks in Vietnam (Nguyen, Fishman, Miatto, & Tanikawa, 2019). Road compositions are verified where information is available as per the study of pothole samples in Chandigarh (Kanoungo, Sharma, Goyal, Kanoungo, & Singh, 2021).

#### Sensitivity

Table S.9: Material intensity of roads from an alternate study in Vietnam (Schiller, Bimesmeier, & Pham, 2020) with comparison of final MIC values to original study values, i.e., MIC values calculated as in Table S.8.

### Summary of key assumptions

Generally, assumptions are made by relating the raw data, i.e., location/type of road, and the measured road width within Google Earth, to the Chandigarh specific hierarchal road network, the recommended land widths for roads (IRC, 2018) and the road composition as per (Nguyen et al., 2019). We assume that all arterial and sub-arterial roads are of the same width, the majority of which are sub-arterial and measure 30m wide. We assume that V1-V4 roads, which relate to the hierarchal road network of Chandigarh named the ‘seven V’s’ (Chandigarh Administration, 2022c; Sarin, 1980), are constructed the same as per the similar visual appearance. V5 roads are assumed to be constructed out of stone as opposed to asphalt and stone, owed to their significantly narrower construction compared to V1-V4 roads and that surface layer is a stone color as opposed to the darker asphalt surface layer from Google Earth imagery. We also assume that service roads are of the same construction as V5. We obtain data from literature for the material intensity of roads and thus assume that the road composition is similar to that in Vietnam as per the study from roads (Nguyen et al., 2019). We omit the use of cycleways, footways, living streets, paths, pedestrian, steps, track and unclassified road types which seem to be partially accounted for relative to the road types.

## Comparison of city-level results to other cities

Table S.10: Data used to calculate MS coefficients for comparison to the present study. Unless referenced otherwise, all values are taken directly from, or calculated using values provided within, the respective study.

Table S.11: Data_from_figure_S.6_in_SI. Comparison of material stock coefficients to other city-level studies

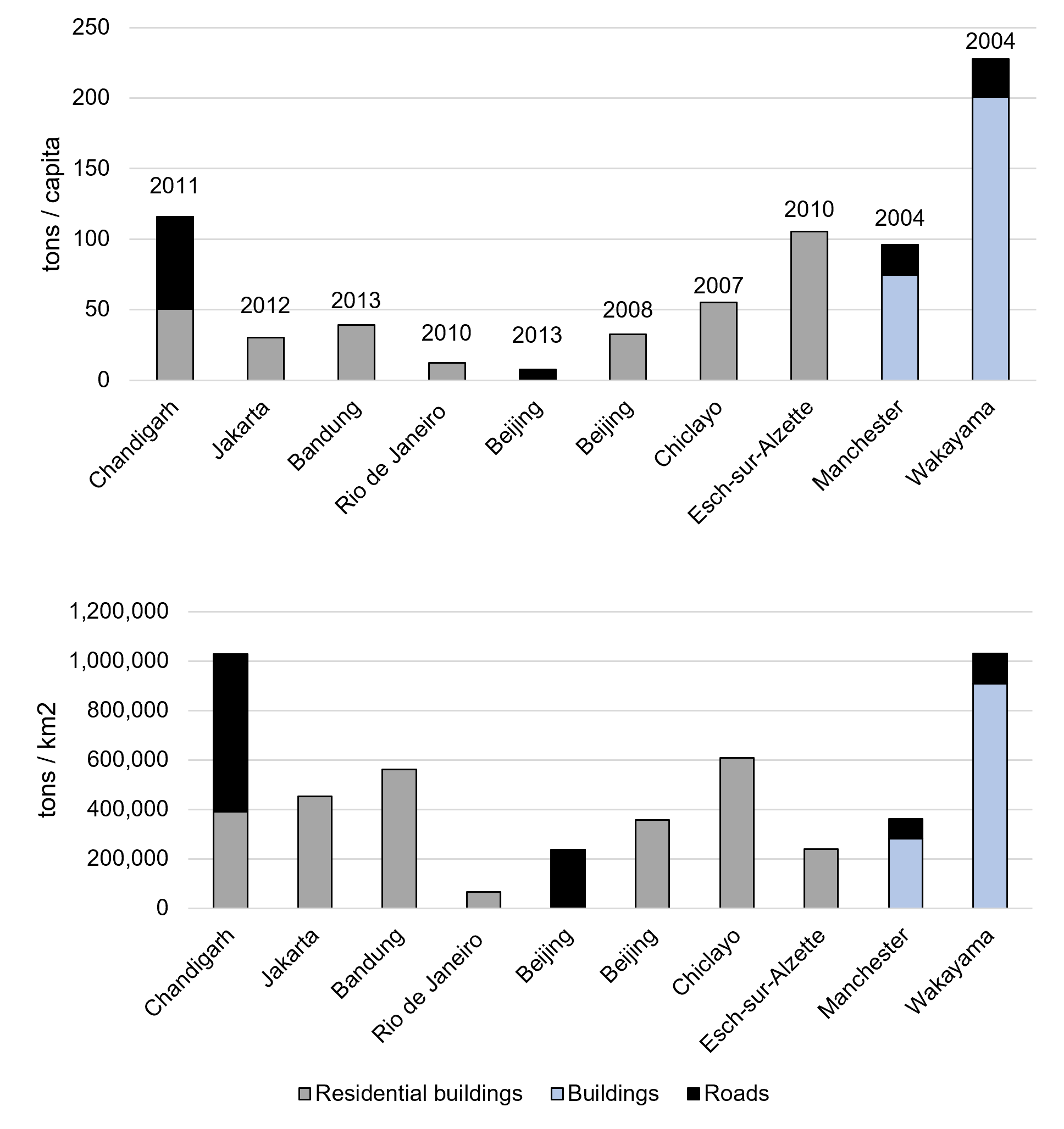


Figure S.6: (Top) Material stock per capita and (bottom) material stock density in comparison to other city-level studies. See Tables S.10 and S.11 for data used.

## A brief discussion on the variation in the accumulation of built environment MS within the wards of Chandigarh

Figure S.6 shows the MS per capita and MS density for a selection of city-level studies from different countries. The findings in comparison to other cities suggest that the unique urban form has driven an unusual accumulation of MS across the city. This has been necessitated by the need to connect people with places and services distributed within and across residential sectors. The results show that the total MS varies among wards, but that relative composition of wards generally remains uniform. This can be largely explained by the homogenous urban form and architectural control as well as variations in the amount of non-residential floor space and the combination of sectors into wards. For example, ward 1 contains a large amount of non-residential floor space compared to others, including a cricket stadium and University, and is comprised of 11 sectors. On the other hand, ward 14 contains only one sector with half of the area dedicated to residential floor space and the other half to a village burial site. This variation highlights the need for further work to quantify non-residential building MS in Chandigarh, as well as for existing studies to account for the sub-city variation in MS to enable an improved understanding and greater comparability of urban form between cities.


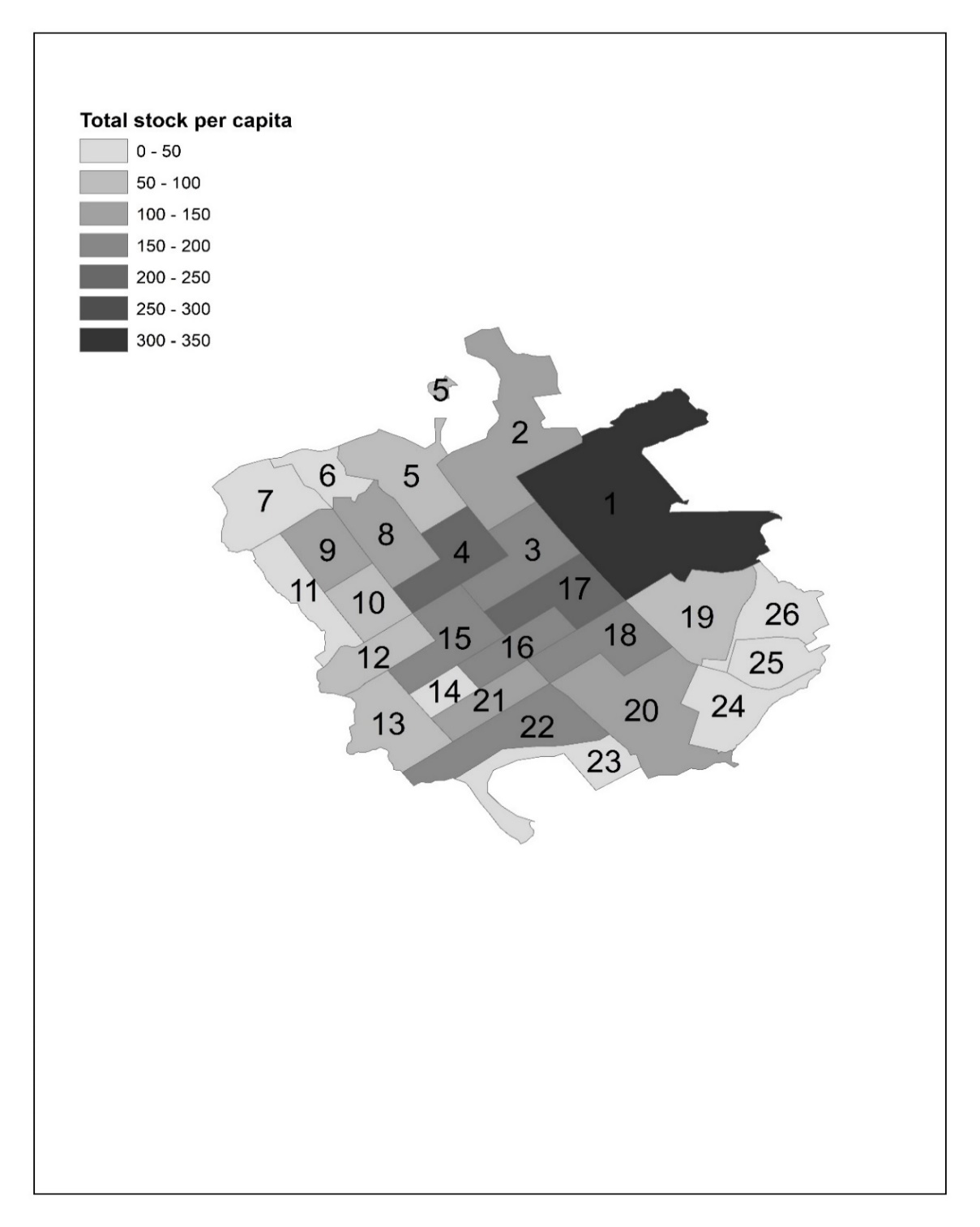

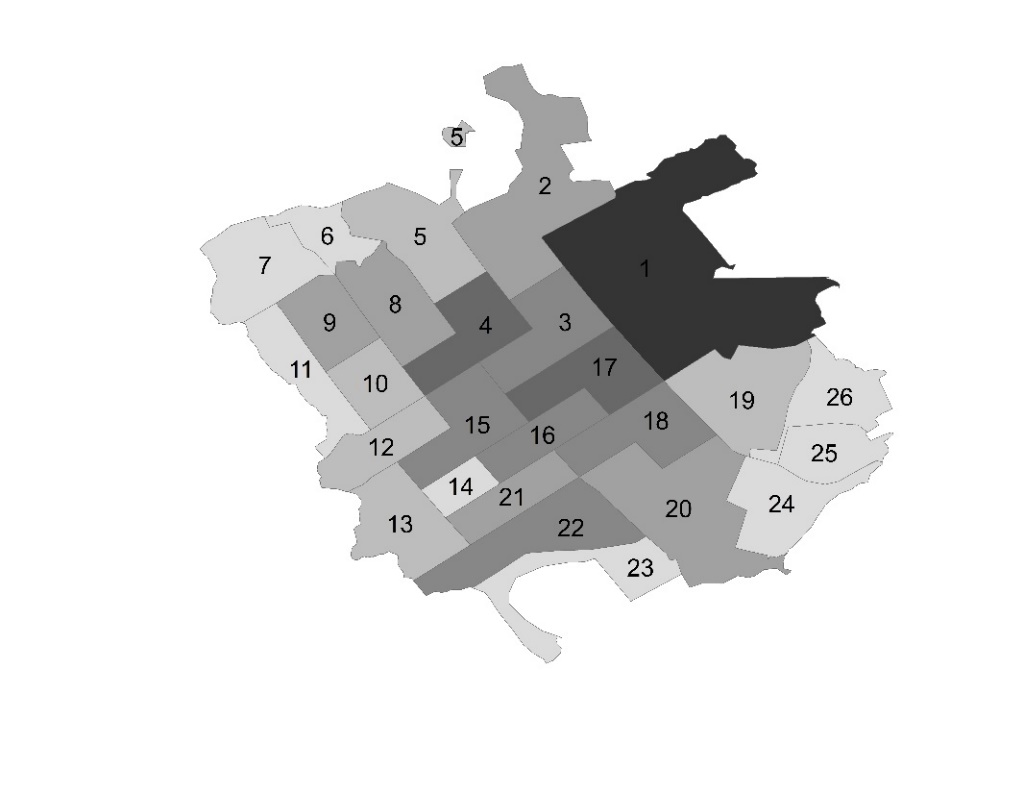


**Total MS per capita** (tons/capita)


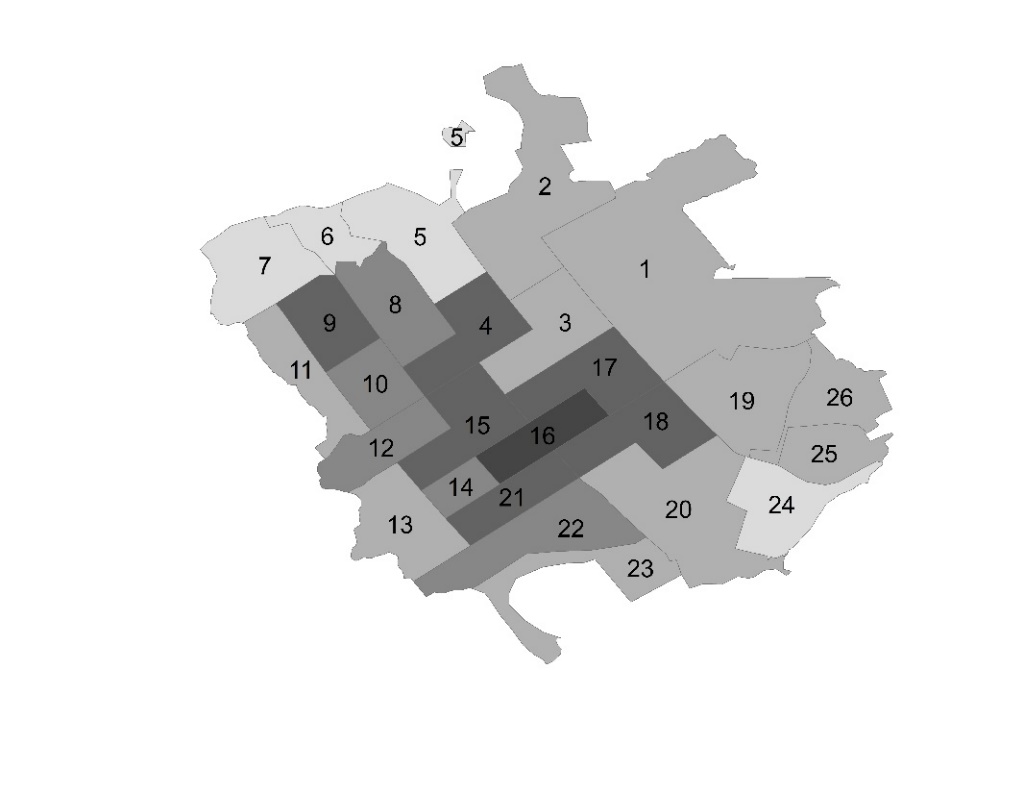


**Total MS per km^2^** (x10^3^ tons/km^2^)


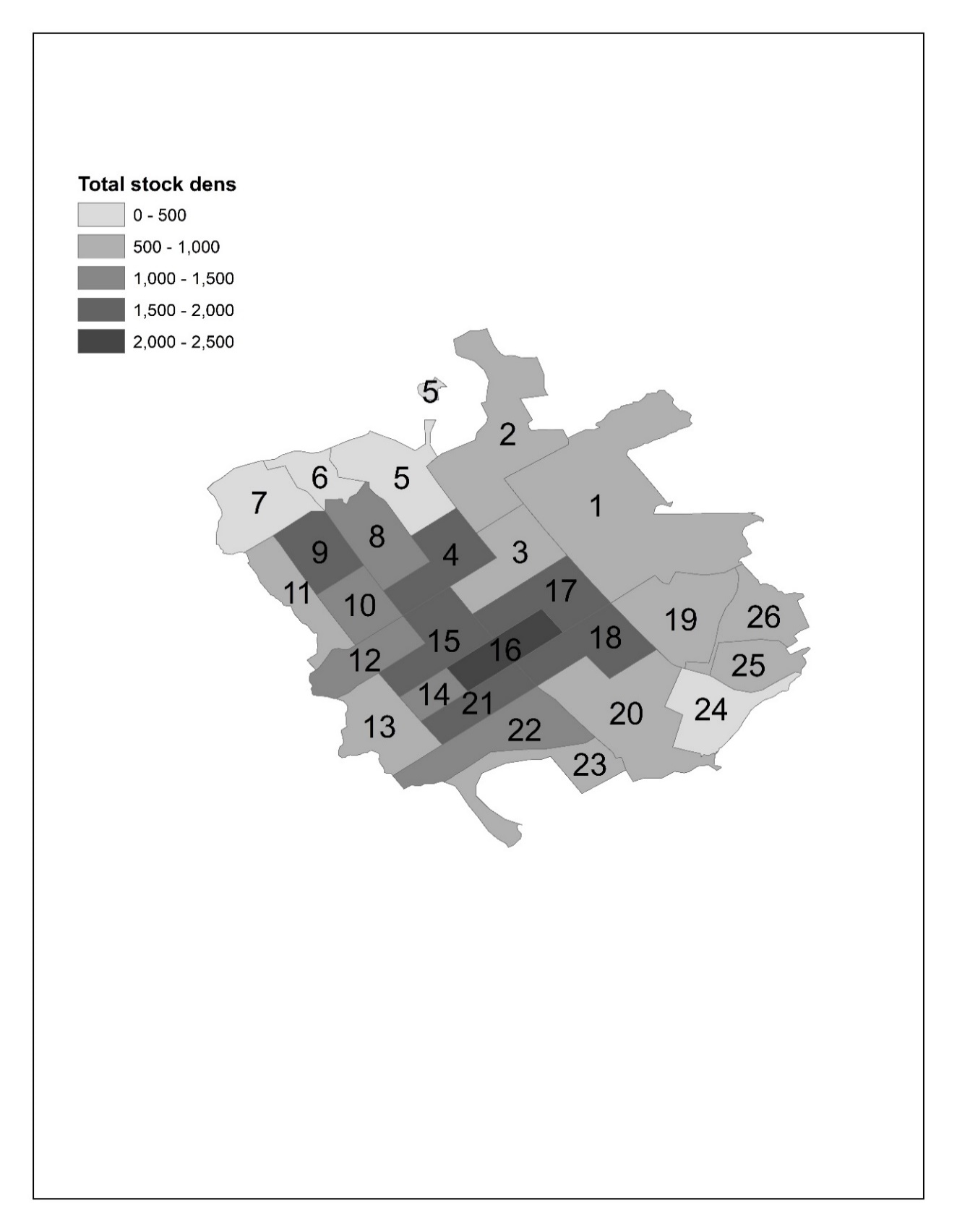

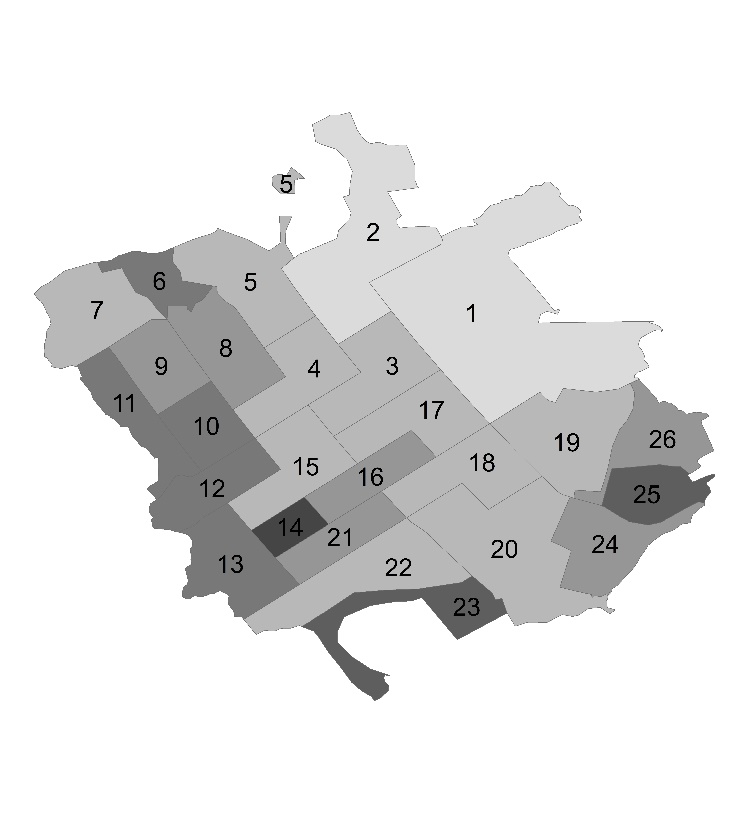

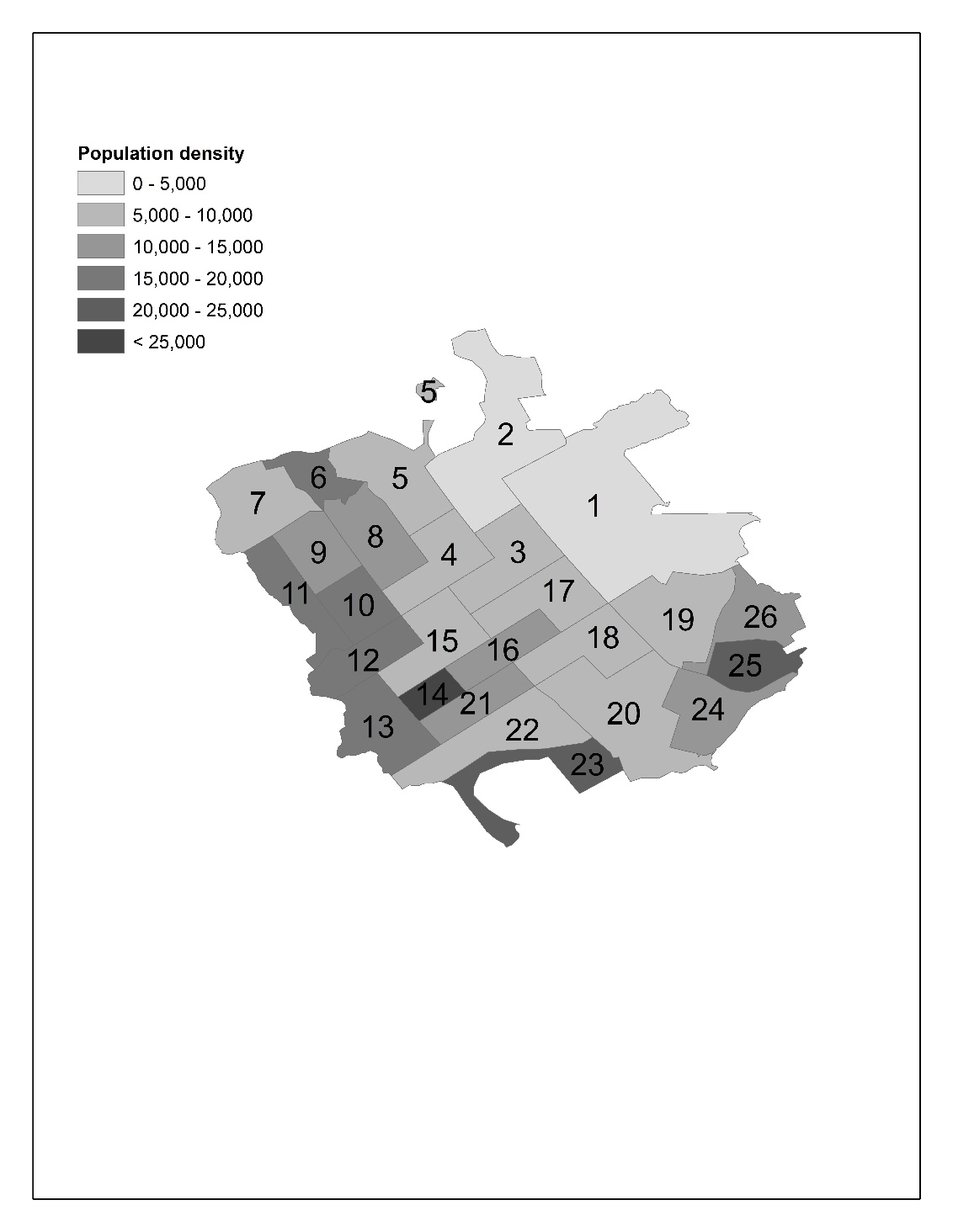


**Population density**

(population/km^2^)

Figure S.7: Total material stock (top) per capita and (middle) per km^2^ in comparison to (bottom) the population density, mapped across the wards of the Municipal Corporation of Chandigarh. The figures show a general pattern that areas with a higher total MS per capita tend to have low population densities, and vice-versa, see Figure 5 of the main text. However, the stock density seems to have little-to-no associated with the population density.

## Chandigarh ward map


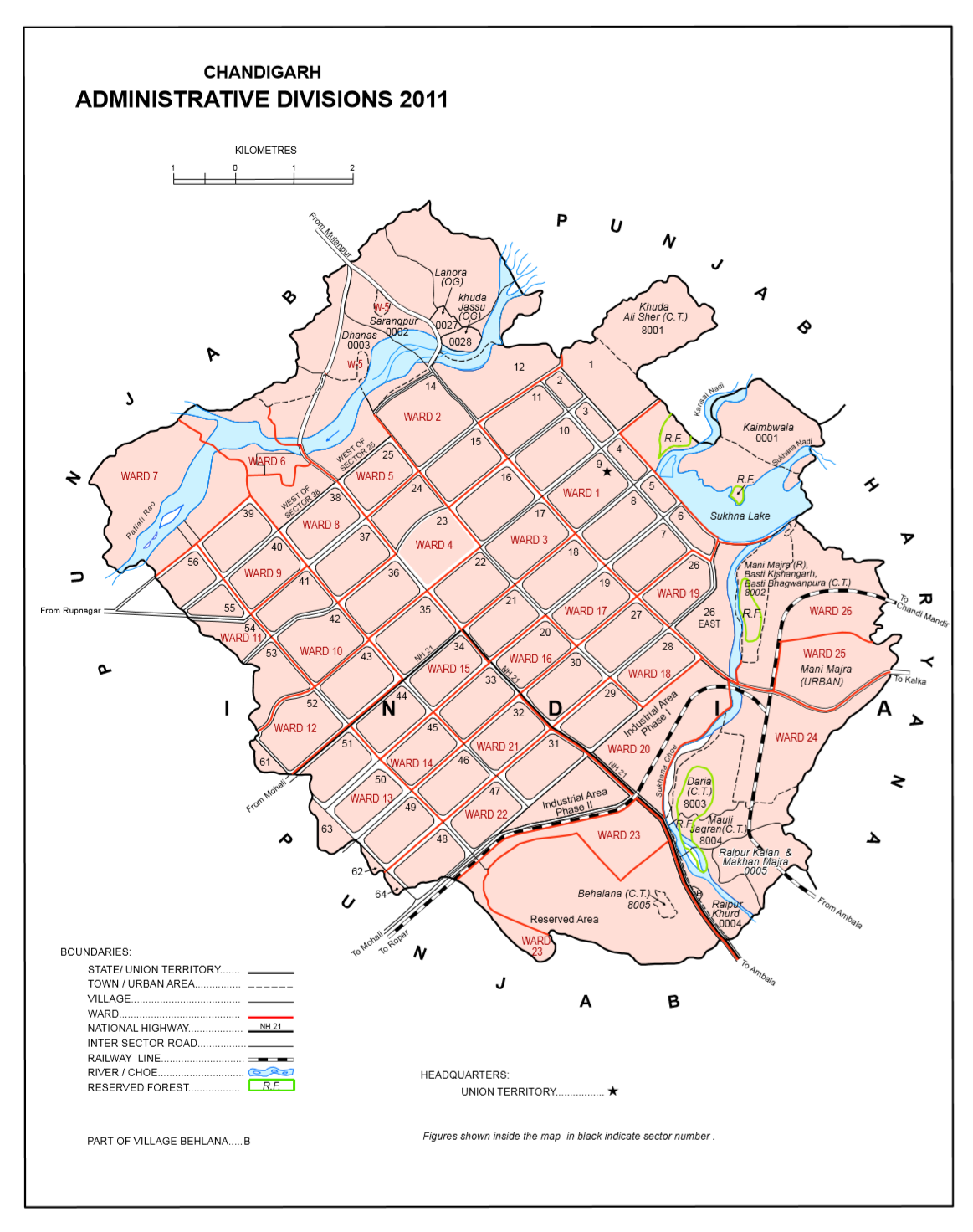


Figure S.8: Map of the administrative divisions of Chandigarh as per the Census of India 2011 (Census of India, 2011)

## References

Bansal, D., & Nandy, P. (2010). *Embodied energy in residential cost effective units (Single Storied) - Up to 50 sqm Plinth area*. (December), 13–14.

Bansal, D., Singh, R., & Sawhney, R. L. (2014). Effect of construction materials on embodied energy and cost of buildings-A case study of residential houses in India up to 60 m2 of plinth area. *Energy and Buildings*, *69*, 260–266. https://doi.org/10.1016/j.enbuild.2013.11.006

Bergsdal, H., Brattebø, H., Bohne, R. A., & Müller, D. B. (2007). Dynamic material flow analysis for Norway’s dwelling stock. *Building Research and Information*, *35*(5), 557–570. https://doi.org/10.1080/09613210701287588

Census of India. (2011). Census of India. Retrieved December 13, 2021, from https://censusindia.gov.in/2011census/hlo/HLO_Tables.html

Chandigarh Administration. (2022a). ARCHITECTURAL CONTROL DRAWINGS | Chandigarh, The official website of the Chandigarh Administration. Retrieved July 13, 2022, from https://chandigarh.gov.in/citizens-facilitation/architectural-control-drawings

Chandigarh Administration. (2022b). CHANDIGARH MASTER PLAN 2031 - The official website of the Chandigarh Administration. Retrieved May 17, 2022, from https://chandigarh.gov.in/chandigarh-master-plan-2031

Chandigarh Administration. (2022c). The official website of the Chandigarh Administration. Retrieved May 17, 2022, from https://chandigarh.gov.in/

Chandigarh Administration. (2022d). Urban Planning - Layout plans. Retrieved July 13, 2022, from https://urbanplanning.chd.gov.in/index.php/home/page/24

Chandigarh Administration. (2022e). ZONING PLANS | Chandigarh, The official website of the Chandigarh Administration. Retrieved July 13, 2022, from https://chandigarh.gov.in/citizens-facilitation/zoning-plans

Chandigarh Housing Board. (2022). Structural Drawings - Chandigarh Housing Board. Retrieved May 24, 2022, from https://chbonline.in/projects/structural-drawings/

Condeixa, K., Haddad, A., & Boer, D. (2017). Material flow analysis of the residential building stock at the city of Rio de Janeiro. *Journal of Cleaner Production*, *149*, 1249–1267. https://doi.org/10.1016/j.jclepro.2017.02.080

Debnath, A., Singh, S. V, & Singh, P. (1995). Comparative assessment of energy requirements for different types of residential buildings in India. In *Energy and Buildings* (Vol. 23).

Directorate of Census Operations, C. (2011). *District Census Handbook Chandigarh*.

García-Torres, S., Kahhat, R., & Santa-Cruz, S. (2017). *Methodology to characterize and quantify debris generation in residential buildings after seismic events*. *117*, 151–159. https://doi.org/10.1016/j.resconrec.2016.11.006

Guo, J., Fishman, T., Wang, Y., Miatto, A., Wuyts, W., Zheng, L., … Tanikawa, H. (2020). Urban development and sustainability challenges chronicled by a century of construction material flows and stocks in Tiexi, China  . *Journal of Industrial Ecology*, jiec.13054. https://doi.org/10.1111/jiec.13054

Guo, Z., Hu, D., Zhang, F., Huang, G., & Xiao, Q. (2014). An integrated material metabolism model for stocks of Urban road system in Beijing, China. *Science of the Total Environment*, *470*–*471*, 883–894. https://doi.org/10.1016/j.scitotenv.2013.10.041

Hu, D., You, F., Zhao, Y., Yuan, Y., Liu, T., Cao, A., … Zhang, J. (2010). Input, stocks and output flows of urban residential building system in Beijing city, China from 1949 to 2008. *Resources, Conservation and Recycling*, *54*(12), 1177–1188. https://doi.org/10.1016/j.resconrec.2010.03.011

Huang, C., Han, J., & Chen, W. Q. (2017). Changing patterns and determinants of infrastructures’ material stocks in Chinese cities. *Resources, Conservation and Recycling*, *123*, 47–53. https://doi.org/10.1016/j.resconrec.2016.06.014

IRC. (2018). *Geometric Design Standards for Urban Roads and Streets*. 2013–2015.

Kanoungo, A., Sharma, U., Goyal, A., Kanoungo, S., & Singh, S. (2021). Assessment of Causes of Pothole Development on Chandigarh Roads. *Journal of The Institution of Engineers (India): Series A*, *102*(2), 411–419. https://doi.org/10.1007/s40030-021-00520-5

Kisan, M., Sangathan, S., & Nehru, J. (1993). Earthquake Resistant Design and Construction of Buildings Code Of Practice. *Bureau of Indian Standars*, *1993*(New Delhi), 1457–1470.

Lanau, M., Liu, G., Kral, U., Wiedenhofer, D., Keijzer, E., Yu, C., & Ehlert, C. (2019). Taking Stock of Built Environment Stock Studies: Progress and Prospects [Review-article]. *Environmental Science and Technology*, *53*(15), 8499–8515. https://doi.org/10.1021/acs.est.8b06652

Mao, R., Bao, Y., Huang, Z., Liu, Q., & Liu, G. (2020). High-Resolution Mapping of the Urban Built Environment Stocks in Beijing. *Environmental Science and Technology*, *54*(9), 5345–5355. https://doi.org/10.1021/acs.est.9b07229

Mastrucci, A., & Rao, N. D. (2019). Bridging India’s housing gap: lowering costs and CO2 emissions. *Building Research and Information*, *47*(1), 8–23. https://doi.org/10.1080/09613218.2018.1483634

Mesta, C., Kahhat, R., & Santa-Cruz, S. (2019). Geospatial Characterization of Material Stock in the Residential Sector of a Latin-American City. *Journal of Industrial Ecology*, *23*(1), 280–291. https://doi.org/10.1111/jiec.12723

Miatto, A., Schandl, H., Wiedenhofer, D., Krausmann, F., & Tanikawa, H. (2017). Modeling material flows and stocks of the road network in the United States 1905–2015. *Resources, Conservation and Recycling*, *127*. https://doi.org/10.1016/j.resconrec.2017.08.024

Nagpure, A. S., Reiner, M., & Ramaswami, A. (2018). Resource requirements of inclusive urban development in India: Insights from ten cities. *Environmental Research Letters*, *13*(2). https://doi.org/10.1088/1748-9326/aaa4fc

Nguyen, T. C., Fishman, T., Miatto, A., & Tanikawa, H. (2019). Estimating the Material Stock of Roads: The Vietnamese Case Study. *Journal of Industrial Ecology*, *23*(3), 663–673. https://doi.org/10.1111/jiec.12773

Ortlepp, R., Gruhler, K., & Schiller, G. (2015). Material stocks in Germany’s non-domestic buildings: a new quantification method. *Building Research and Information*, *44*(8), 840–862. https://doi.org/10.1080/09613218.2016.1112096

Ortlepp, R., Gruhler, K., & Schiller, G. (2018). Materials in Germany’s domestic building stock: calculation model and uncertainties. *Building Research and Information*, *46*(2), 164–178. https://doi.org/10.1080/09613218.2016.1264121

Praseeda, K. I., Reddy, B. V. V., & Mani, M. (2016). Embodied and operational energy of urban residential buildings in India. *Energy and Buildings*, *110*, 211–219. https://doi.org/10.1016/j.enbuild.2015.09.072

Ram, V. G., & Kalidindi, S. N. (2017). Estimation of construction and demolition waste using waste generation rates in Chennai, India. *Waste Management and Research*, *35*(6), 610–617. https://doi.org/10.1177/0734242X17693297

Ramesh, T., Prakash, R., & Kumar Shukla, K. (2013). Life Cycle Energy Analysis of a Multifamily Residential House: A Case Study in Indian Context. *Open Journal of Energy Efficiency*, *02*(01), 34–41. https://doi.org/10.4236/ojee.2013.21006

Rao, N. D., Min, J., & Mastrucci, A. (2019). Energy requirements for decent living in India, Brazil and South Africa. *Nature Energy*, *4*. https://doi.org/10.1038/s41560-019-0497-9

Sarin, M. (1980). Chandigarh: Progress and problems of a great experiment. *The Round Table*, *70*(279), 299–304. https://doi.org/10.1080/00358538008453467

Schiller, G., Bimesmeier, T., & Pham, A. T. V. (2020). Method for Quantifying Supply and Demand of Construction Minerals in Urban Regions-A Case Study of Hanoi and Its Hinterland. *Sustainability*. https://doi.org/10.3390/su12114358

Schiller, G., Miatto, A., Gruhler, K., Ortlepp, R., Deilmann, C., & Tanikawa, H. (2018). Transferability of Material Composition Indicators for Residential Buildings: A Conceptual Approach Based on a German-Japanese Comparison. *Journal of Industrial Ecology*, *23*(4). https://doi.org/10.1111/jiec.12817

Surahman, U., Higashi, O., & Kubota, T. (2017). Evaluation of current material stock and future demolition waste for urban residential buildings in Jakarta and Bandung, Indonesia: embodied energy and CO2 emission analysis. *Journal of Material Cycles and Waste Management*, *19*(2). https://doi.org/10.1007/s10163-015-0460-1

Tanikawa, H., Fishman, T., Okuoka, K., & Sugimoto, K. (2015). The weight of society over time and space: A comprehensive account of the construction material stock of Japan, 1945-2010. *Journal of Industrial Ecology*, *19*(5), 778–791. https://doi.org/10.1111/jiec.12284

Tanikawa, H., & Hashimoto, S. (2009). Urban stock over time: Spatial material stock analysis using 4d-GIS. *Building Research and Information*, *37*(5–6), 483–502. https://doi.org/10.1080/09613210903169394

Vengala, J., Ramesh, K., Dharek, M. S., Krishna, B., & Kumar, S. (2021). Embodied energy and operational energy computations for a typical G+3 residential building in Vijayawada city of Andhra Pradesh, India. *International Journal of Advanced Technology and Engineering Exploration*, *8*(81), 2394–7454. https://doi.org/10.19101/IJATEE.2021.874169

Wiedenhofer, D., Steinberger, J. K., Eisenmenger, N., & Haas, W. (2015). Maintenance and Expansion: Modeling Material Stocks and Flows for Residential Buildings and Transportation Networks in the EU25. *Journal of Industrial Ecology*, *19*(4), 538–551. https://doi.org/10.1111/jiec.12216
